# Transcriptional Activation, Deactivation and Rebound Patterns in Cortex, Hippocampus and Amygdala in Response to Ketamine Infusion in Rats

**DOI:** 10.3389/fnmol.2022.892345

**Published:** 2022-05-30

**Authors:** Jenny J. Kim, Matthew R. Sapio, Fernando A. Vazquez, Dragan Maric, Amelia J. Loydpierson, Wenting Ma, Carlos A. Zarate, Michael J. Iadarola, Andrew J. Mannes

**Affiliations:** ^1^Department of Perioperative Medicine, Clinical Center, National Institutes of Health, Bethesda, MD, United States; ^2^Flow and Imaging Cytometry Core Facility, National Institute of Neurological Disorders and Stroke, National Institutes of Health, Bethesda, MD, United States; ^3^Experimental Therapeutics and Pathophysiology Branch, National Institute of Mental Health, National Institutes of Health, Bethesda, MD, United States

**Keywords:** ketamine, transcriptomic (RNA-Seq), molecular pharmacology, RNA-Seq, anesthesia, systems pharmacology, neuroscience, neuro-pharmacology

## Abstract

Ketamine, an *N*-methyl-D-aspartate (NMDA)-receptor antagonist, is a recently revitalized treatment for pain and depression, yet its actions at the molecular level remain incompletely defined. In this molecular-pharmacological investigation in the rat, we used short- and longer-term infusions of high dose ketamine to stimulate neuronal transcription processes. We hypothesized that a progressively stronger modulation of neuronal gene networks would occur over time in cortical and limbic pathways. A continuous intravenous administration paradigm for ketamine was developed in rat consisting of short (1 h) and long duration (10 h, and 10 h + 24 h recovery) infusions of anesthetic concentrations to activate or inhibit gene transcription in a pharmacokinetically controlled fashion. Transcription was measured by RNA-Seq in three brain regions: frontal cortex, hippocampus, and amygdala. Cellular level gene localization was performed with multiplex fluorescent *in situ* hybridization. Induction of a shared transcriptional regulatory network occurred within 1 h in all three brain regions consisting of (a) genes involved in stimulus-transcription factor coupling that are induced during altered synaptic activity (immediate early genes, IEGs, such as c-Fos, 9–12 significant genes per brain region, *p* < 0.01 per gene) and (b) the Nrf2 oxidative stress-antioxidant response pathway downstream from glutamate signaling (Nuclear Factor Erythroid-Derived 2-Like 2) containing 12–25 increasing genes (*p* < 0.01) per brain region. By 10 h of infusion, the acute results were further reinforced and consisted of more and stronger gene alterations reflecting a sustained and accentuated ketamine modulation of regional excitation and plasticity. At the cellular level, *in situ* hybridization localized up-regulation of the plasticity-associated gene Bdnf, and the transcription factors Nr4a1 and Fos, in cortical layers III and V. After 24 h recovery, we observed overshoot of transcriptional processes rather than a smooth return to homeostasis suggesting an oscillation of plasticity occurs during the transition to a new phase of neuronal regulation. These data elucidate critical molecular regulatory actions during and downstream of ketamine administration that may contribute to the unique drug actions of this anesthetic agent. These molecular investigations point to pathways linked to therapeutically useful attributes of ketamine.

## Introduction

Ketamine is a phencyclidine analog that has important clinical uses as an analgesic, an anesthetic, and an antidepressant. Introduced as a general anesthetic in the 1960s, its use as an analgesic has gained renewed attention, reflecting the need to utilize non-opioid alternatives in the management of acute and chronic pain. Despite strong interest in the use of ketamine in clinical practice, either alone or in combination with other analgesics (Kharasch and Clark, 2021; Murphy et al., 2021) many questions remain regarding the molecular and systems-level consequences of ketamine administration. Although ketamine exerts its primary pharmacodynamic effects by acting as an *N*-methyl-D-aspartate Receptor (NMDAR) antagonist, the ubiquitous nature of the NMDAR throughout the central nervous system and abundant evidence for non-NMDAR ketamine actions (Roth et al., 2013; Maeng et al., 2008; Zanos et al., 2016; Yao et al., 2018) necessitate additional comprehensive molecular evaluations. Several recent studies have investigated ketamine’s synaptic actions and molecular effects, leading to new ideas about how this drug acts (Zanos and Gould, 2018; Duman et al., 2016; Maeng et al., 2008; Moda-Sava et al., 2019; Bagot et al., 2017; Upton et al., 2020). Briefly, ketamine induces a paradoxical excitatory state as supported by increased gamma band power on quantitative electroencephalography (Sanacora et al., 2014; Nugent et al., 2019b), increased glutamate neurotransmission (Abdallah et al., 2018), and increased hippocampal synaptic potentiation (Nosyreva et al., 2013). The etiology of the excitatory action is not certain, although it has been attributed to cortical interneuron-mediated network disinhibition, antagonism of extrasynaptic GluN2B-containing NMDARs (Miller et al., 2014), and drug metabolite actions on α-amino-3-hydroxy-5-methyl-4-isoxazole-propionic acid receptors (AMPARs; Zanos et al., 2016). Ketamine administration acutely activates a cascade of downstream synaptic regulators such as brain-derived neurotrophic factor (BDNF; Autry and Monteggia, 2012; Duman et al., 2016; Laje et al., 2012), mammalian target of rapamycin (mTOR; Li et al., 2010), and eukaryotic elongation factor 2 kinase (eEF2K; Autry et al., 2011; Zanos et al., 2016; Tornese et al., 2019). These alterations, which drive synaptic rearrangements (Autry et al., 2011; Li et al., 2010; Duman et al., 2016) likely underlie long-term therapeutic effects such as remission of depression symptoms and the potential for modification of chronic pain syndromes (Moda-Sava et al., 2019; Zarate et al., 2006; Berman et al., 2000; Autry et al., 2011; Murrough et al., 2013; Fava et al., 2018; Clark, 2020). We hypothesized that paradoxical elevation of neuronal activity-coupled transcriptional signaling pathways would distinguish intravenous ketamine infusion from other anesthetic agents which are marked by decreases in these genes.

The present study investigates ketamine’s molecular and neuronal mechanisms of action at the level of transcriptional modulation occurring acutely at 1 h, and after 10 h of continuous infusion as well as more sustained alterations measured at 24 h after cessation of the drug. These time points were chosen for their relationship to therapeutic usage (1 h) and biochemical considerations of the rate of mRNA turnover needed to reveal modification of transcriptional processes (10 h; Singer and Penman, 1972). We hypothesized that we could capture the initiation phase at 1 h and the transition to effector genes at 10 h. The considerations for driving transcriptional machinery also influenced the choice of dose, which was high enough to induce a consistent, sustained anesthetic plane marked by loss of righting reflex and response to noxious stimuli. A second reason for developing the infusion paradigm is that previous studies had difficulty detecting robust molecular modifications with dosing parameters that typically model the shorter duration administration times characteristic of antidepressant and pain interventional clinical paradigms. Therefore, one aim was to first investigate a highly impactful dose to establish a foundational basis before examining more subtle changes occurring with lower dosing paradigms.

## Materials and Methods

### Methodological Overview

**FIGURE 1:**
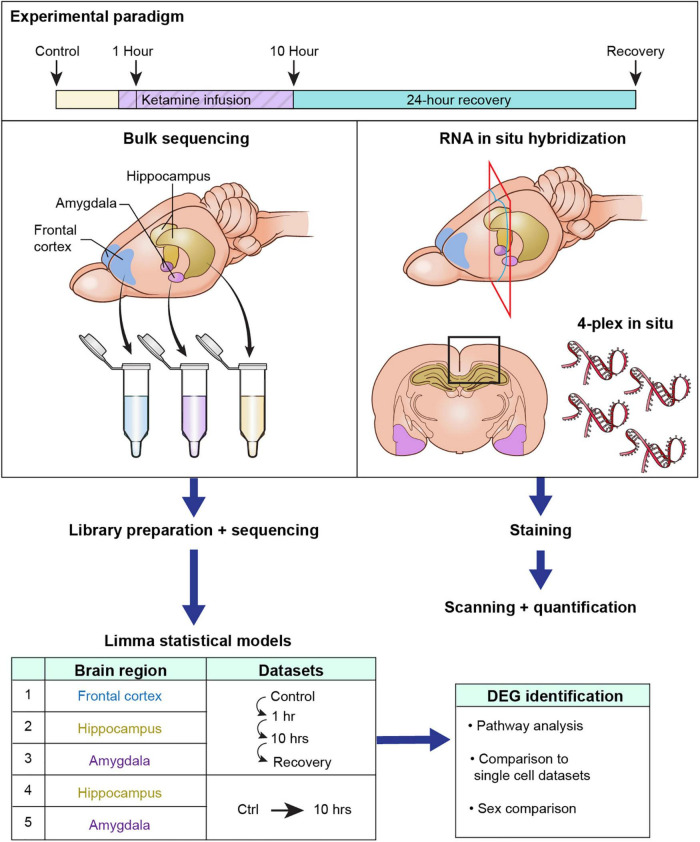
Diagrammatic summary of experimental design and analyses. This study assessed the gene expression changes in brain in response to intravenous ketamine in rats with an implanted femoral vein catheter. Rats were infused with ketamine, and brain tissue was harvested at 1, 10, or 24 h after cessation of a 10 h infusion. Catheterized controls were used as a baseline comparator. The detailed breakdown of *N* per animal per group is shown in Table 1. The brain was sub-dissected rapidly into frontal cortex (the entire frontal pole anterior to the forceps minor of the corpus callosum), amygdala, and hippocampus. Bulk RNA-Seq was performed on each homogenized brain region at each time point. Tissue was also collected for multiplex *in situ* hybridization to examine anatomical distribution of select differential genes quantitatively and qualitatively. After sequencing, 5 statistical models were built inside the limma package (considering each brain region x sex as a separate experiment). The significant genes from each comparison were considered. After identification of differential genes (DEGs), pathway analysis was performed to identify groups of genes changing in concert as part of biological processes. Single cell datasets from other previously published studies were referenced to identify the cellular origin of gene changes within the homogenate. Finally, we examined male and female rats to validate that female rats reproduced some of the most important findings from the experiment.

Due to the complex nature of the present report this section summarizes the overview of how experiments were conducted (Figure 1). Tissue collection was performed at control (ketamine naïve time points) or after 1 h or 10 h of ketamine infusion. A final group was collected at 24 h after cessation of a 10 h infusion (recovery). These time points were selected based on known dynamics in mRNA turnover rates. Given the half-life of the average mRNA, the minimum time point to observe a transcriptional decrease using RNA-Seq is about 10 h, which corresponds to ∼4 half lives (Singer and Penman, 1972). Conversely, induction of immediate early genes (IEGs) can be observed by 1 h. This is diagrammatically explained in Supplementary Figure 1. At each time point brains were collected and subdivided into frontal cortex, hippocampus and amygdala based on anatomical landmarks. While several subregions of these broad anatomical divisions have been specifically implicated in ketamine responses, in particular the ventral vs. dorsal subregions of the hippocampus (Carreno et al., 2016; Yamada and Jinno, 2019), the present study focused on large areas of brain. This approach will capture broad gene expression changes and can be refined in subsequent studies focused on specific subareas. Separate cohorts of animals were used for experiments where brain regions were homogenized vs. those in which tissue was fixed and stained. The same facility, ages and experimental procedures (described below) were chosen for both methodologies. After sequencing models were built to find significant genes over time using the limma package to identify differentially expressed genes. Five models were built: Male Frontal Cortex (Control, 1 h, 10 h, Recovery); Male Hippocampus (Control, 1 h, 10 h, Recovery); Male Amygdala (Control, 1 h, 10 h, Recovery); Female Hippocampus (Control, 10 h); Female Amygdala (Control, 10 h). Significant genes from each of these 5 comparisons were used to generate the figures as described below.

### Animal Usage and Time Point Selection

The Institutional Animal Care and Use Committee of the Clinical Center, National Institutes of Health (Bethesda, MD) approved all experiments. Male and female Sprague Dawley rats (250–400 g) were implanted unilaterally with a femoral vein catheter at Charles River Laboratories (Raleigh, NC). Rats were infused between post-operative day 4 and 7. Catheterized animals were randomly selected for ketamine infusion or assigned to the control group and infused for either 1 or 10 h with intravenous ketamine.

Within each cohort of animals used for comparisons, animals were born at the same time and were approximately the same weight. Implanted animals were individually housed after surgery with 12 h light-dark cycles and fed *ad libitum*. Behavioral testing and monitoring were conducted during the animal’s light cycle. Animal cages were furnished with a plastic tunnel for enrichment.

### Chronic Catheter Implantation

The femoral vein catheter was inserted and secured according to standard operating procedures by Charles River Laboratories. The free end of the catheter was tunneled subcutaneously and attached to a transcutaneous access port (Instech model #VABR1B/22, Plymouth Meeting, PA) located in the intrascapular region. Catheterized animals arrived at the National Institutes of Health (NIH) between post-operative day 3 and 5. Upon arrival, animals were outfitted with a magnetic metal cap to prevent contamination of the transcutaneous access port (Instech Model No. #VABRC, Plymouth Meeting, PA). The catheter was periodically flushed with 100–200 μl heparinized saline (500 IU/mL) to maintain patency. Pre-operatively, animals were injected with 5–10 mg/kg enrofloxacin subcutaneously. This dose was also administered for 2 days postoperatively. Intraoperatively, analgesia was administered by injection of 3–5 mg/kg carprofen subcutaneously. Postoperatively, animals were given oral carprofen as MediGel CGP (Clear H2O Inc., Westbrook, ME). Each pair of rats were given one 2 oz. cup per day per the manufacturer’s instructions. Animals did not show signs of distress postsurgically.

### Experimental Design

Male animals were divided into 3 experimental groups (1 h: *n* = 6, 10 h: *n* = 7, 10 h with 24 h recovery: *n* = 6) and a control group (*n* = 7). Catheterized animals in the control group were euthanized between post-operative day 4 and 7 relative to the catheterization. Catheterized controls did not receive an infusion due to the apparent stress that awake infusions caused using this catheter system. However, the longitudinal design allows for degrees of onset (an increasing degree from 1 to 10 h) and a return to baseline (by 24 h) which internally controls for the catheterization and infusion procedure. Animals in the recovery group were administered a 10 h ketamine infusion, allowed 24 h for recovery, and then euthanized. In female rats, we reproduced the control and 10 h infusion groups in order to investigate the effect of sex on transcriptomic alterations after ketamine infusion (*n* = 5 each). The 10 h infusion produced the most robust regulatory modulation and was hypothesized to provide the most assay sensitivity for distinguishing a difference due to sex. Numbers of animals required for experiments were estimated by considering power requirements for the RNA-Seq analyses based on a previous study from our laboratory (Raithel et al., 2018), and this number was estimated to be greater than that for either *in situ* hybridization or behavioral assessments. Note that in addition to being consistent with our previous reports, the group sizes used in the present study are estimated to be adequate for most gene changes in studies of laboratory animals using RNA-Seq (Schurch et al., 2016).

### Titration of Intravenous Infusion for Induction and Maintenance

Administration of ketamine was achieved through a femoral vein infusion line (Instech model #KVABR1T/22) designed to fit the magnetic vascular access button.

**FIGURE 2:**
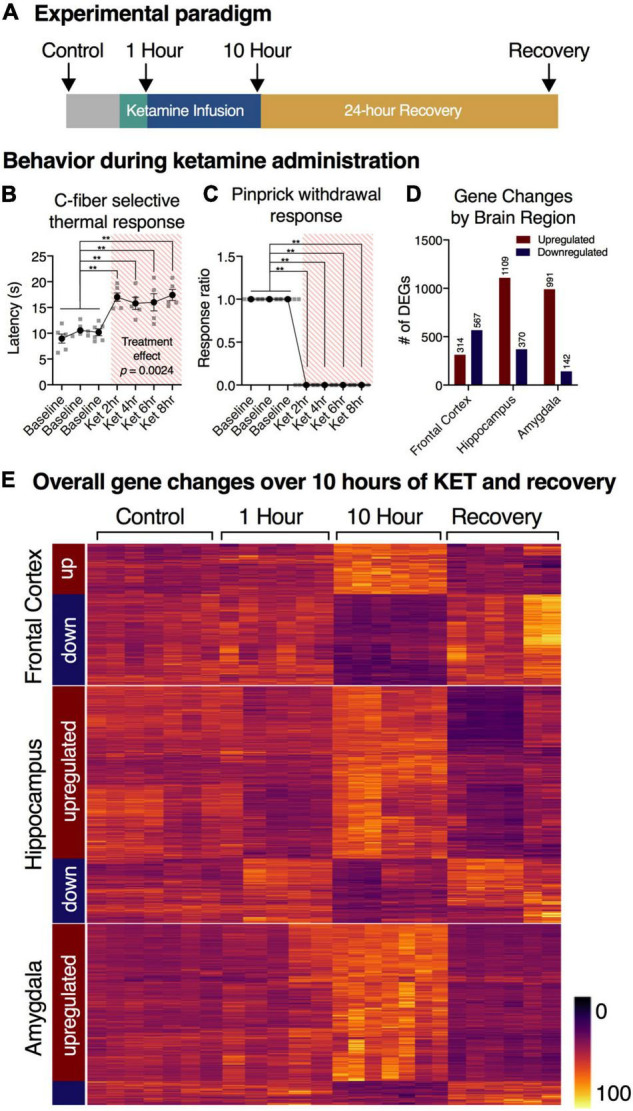
Experimental overview and summary of RNA-Seq findings. **(A)** Timeline of experimental procedures. RNA-Seq was performed on the frontal lobe, hippocampus, and amygdala from sham operated controls (*n* = 7), 1 h infusion (*n* = 6), 10 h infusion (*n* = 7), and 10 h infusion + 24 h recovery (*n* = 6). **(B,C)** Response to thermal C-fiber activation, and mechanical pinprick are attenuated during the ketamine infusion. These responses indicate blockade of pain sensation in both modalities. Statistics were performed using a mixed-effects model (thermal), or binomial test (pinprick) with Holm-Šidák corrections (*n* = 6). Error bars show standard error of the mean. For pinprick response **(C)**, no variance was observed. **(D)** Total number of genes regulated as assessed by RNA-Seq. Differentially expressed genes (DEGs) were defined as those with adjusted *p*-value < 0.01 and had > 40% expression ratio at 10 sFPKM (variable expression ratio threshold, see “Materials and Methods”). Datasets were analyzed using statistical package limma. The total number of genes meeting threshold were as follows: Frontal Cortex: 314 up, 567 down; Hippocampus: 1109 up, 370 down; Amygdala: 991 up, 142 down. Note that in the amygdala and hippocampus there were more upregulated genes, whereas in the frontal cortex roughly equal numbers of significantly up- and downregulated genes were observed. **(E)** Clustering of gene changes by brain region. Note that the most prominent gene changes occurred at the 10 h time point, and the genes clustered according to direction at 10 h.

Loss of righting reflex was used as the primary behavioral endpoint to establish the ketamine dose. The *minimum* dose required to induce and maintain immobility was selected. Rats were gently restrained manually and administered 20–30 mg/kg of ketamine and titrated by 10 mg/kg increments. The use of gaseous anesthesia was avoided during ketamine induction, in part due to concerns regarding synergistic sedating effects of these drugs, leaving them to be contraindicated in combination with ketamine. Shortly after bolus induction, in the first 2 h of infusion, continuous adjustment of dosage was required to maintain the sedative plane. After the initial 2 h, males received 0.4–1.1 mg/kg/min while females received 0.2–0.5 mg/kg/min ketamine on average. These infusion values delineate the range of intermittent adjustments, but no animal received a sustained infusion of 1.1 mg/kg/hr. Thus, the dose ranges listed reflect continuous adjustment, and not a large inter-animal difference as over the 10-h period the dose was adjusted to establish a steady sedative plane. Supporting this idea is that the physiological variables of oxygen saturation, blood pressure and body temperature remained constant over the 10 h period (Supplementary Figure 2). We observed that a higher dose was needed to reach the same loss of righting reflex in the males than the females. We also observed that loss of righting reflex occurred without producing complete loss of spontaneous body movements or shallow breathing. The spontaneous movements during ketamine administration included mild back and forth head movements and whisking of vibrissal hairs and apparent sniffing behaviors. These movement occurred despite suppression of responses to noxious stimuli. Another parameter used in determining the sedative plane was to titrate the lightest dose possible to block withdrawal to hind paw pinch with toothed forceps, pin prick, and thermal stimulation (see below, and Figure 2).

### Monitoring of Physiological Parameters During Ketamine Infusion

Electrocardiography, pulse oximetry, respiratory rate, and rectal temperature were monitored in a sentinel animal throughout the infusion (Supplementary Figure 2), as follows. Rats were continuously monitored using a Philips Intellivue MP70 patient monitor attached to SpO2 sensor (Covidien OxiMax Adhesive; Dublin, Ireland), neonatal pre-wired EKG monitoring electrodes (3M*™* Red Dot*™*; St Paul, MN, United States) and a rectal temperature probe (Covidien 400 Series General Purpose Temperature Probe, 9Fr; Dublin, Ireland). Body temperature was maintained at 37–39°C using a perfused water pad underneath the animals (T/Pump, Stryker; Kalamazoo, MI, United States). Animals under ketamine infusion remained hemodynamically stable with blood oxygenation > 95% and in sinus rhythm. Total intravenous fluids administered did not exceed the recommended daily maintenance amount of 80 mL/kg/day (Waynforth and Flecknell, 1992). Physiological monitoring of the female group during ketamine infusion was also performed and parameters were within normal limits.

### Nociceptive Testing

In order to ascertain whether the infusion parameters produced a stable alteration in nociceptive sensory processing, we administered thermal and mechanical nociceptive stimuli to the hind paw at 2 h intervals during the 10 h infusion period. For comparison, awake ketamine-naïve catheterized rats, were tested to establish a baseline for nociceptive sensitivity measurements. Awake, unrestrained animals were habituated to a plastic enclosure on a glass platform on 3 consecutive days. On days 2 and 3 of the habituation period animals were tested for thermal nociceptive withdrawal latency to acclimate them to the thermal test itself. Following acclimation, withdrawal responses to both pin prick and thermal heat were recorded in an additional 3 test sessions given over 2 days. Both hind paws were tested in each animal. Thermal stimuli were delivered by an infrared diode laser (LASS-10 M; LasMed, Mountain View, CA, United States) with an output wavelength of 980 nm as previously described (Mitchell et al., 2014; Sapio et al., 2019). C-fiber mediated responses were evoked by continuous application of a 5 mm diameter beam at 650 mA. Responses were recorded as the time until paw withdrawal (withdrawal latency), with a cutoff at 25s if no response occurred. Ketamine infused animals were tested with identical laser parameters, and consistent laser to paw distance. Pinprick stimulation was applied to the plantar surface with a hand-held dissecting pin to assay mechano-sensory responsiveness (Blivis et al., 2017). The dependent measure recorded was a simple binary outcome, withdrawal vs non-withdrawal.

### Tissue Preparation and RNA Purification, Library Preparation and Next-Generation Sequencing

Animals were decapitated at the end of the ketamine infusion while still anesthetized; the recovery animals were anesthetized by brief exposure to 5% isoflurane prior to decapitation. Tissues from catheterized controls or 10 h ketamine infused animals were collected on the same day. Briefly, whole brain was removed from the skull and rinsed with ice cold PBS to remove any adherent blood. Frontal cortex was isolated anterior to the corpus callosum after first removing the olfactory bulbs which were blunt dissected and cut away anterior to the olfactory tubercle. Whole hippocampus was harvested via blunt dissection from the dorsal midline using curved forceps. The hippocampus was visually inspected to ensure that neither cortex nor choroid plexus were included with the hippocampus. Amygdala and adjacent periamygdaloid cortex were removed from a slice of tissue. The posterior border was the temporal tip of the hippocampus (∼−3.8 mm from bregma; Paxinos and Watson, 2014), the anterior border was denoted by the posterior optic chiasm (∼−1.8 mm from bregma), and the dorsal border was the rhinal sulcus (Naranjo et al., 1986). Fully dissected samples were rinsed with ice cold PBS and flash frozen on dry ice in Lysing Matrix D tubes (MP Biomedicals, United States). Tissue was stored at −80°C until extraction.

Samples were processed for RNA extraction with controls and experimental samples extracted at the same time to avoid batch effects. Total RNA was extracted using an amended protocol based on the RNeasy Lipid Tissue Mini Kit with optional DNase digestion (Qiagen, United States). Briefly, phenol/guanidine thiocyanate-based QIAzol lysis reagent (Qiagen) was added to the tissue and bead matrix on dry ice. Subsequently, a second aliquot of room temperature QIAzol was added, and the total mixture was allowed to thaw to a frozen slurry, which was immediately homogenized using the Fast Prep-24 Homogenizer (MP Biomedicals, United States). Chloroform was added and phases were separated by centrifugation. The aqueous layer was retrieved and combined with an equal volume of ice-cold 70% ethanol and added to the RNeasy Mini Spin Column (Qiagen, United States). The column was washed with buffer RW1 and incubated on-column with RNase-Free DNase (Qiagen, United States) at room temperature for 15 minutes. The columns were washed according to standard procedures with an additional wash of RW1 and RPE solutions from the Qiagen kit to increase stringency. RNA was eluted from the column in RNase-free water, aliquoted, and kept at −80°C. RNA integrity and purity were evaluated by gel electrophoresis on a 2100 Bioanalyzer with RNA 6000 Nano Kit (Agilent Technologies, United States) and NanoDrop 2000 Spectrophotometer (Thermo Scientific, United States), respectively. All samples had an RNA integrity score greater than or equal to 8.4.

### Library Preparation and Next-Generation Sequencing

Library preparation and sequencing were conducted at the National Institutes of Health Intramural Sequencing Center. Briefly, Poly-A^+^ selected, stranded mRNA libraries were constructed from 1 μg total RNA using the Illumina TruSeq Stranded mRNA Sample Prep Kits according to manufacturer’s instructions except where noted. Amplification was performed using 10 cycles to minimize the risk of over-amplification. Unique dual-indexed barcode adapters were applied to each library. Libraries were pooled in an equimolar ratio for sequencing. The pooled libraries were sequenced on one lane of an S4 flow cell on a NovaSeq 6000 using version 1 chemistry to achieve a minimum of 46 million 150 base read pairs. The data was processed using RTA version 3.3.3 and BWA-0.7.12. Sequence data (FASTQ files) are available for download through the Sequence Read Archive under BioProject PRJNA607336.

### Alignment, Quantification, and Statistical Analyses of RNA-Seq Data

Datasets were aligned using MAGIC software (version magic.2017_06_19; Wang et al., 2014; Zhang et al., 2015) with a genomic target based on Rn6 (rat) with RefSeq annotations. Quality control was performed in MAGIC, and failure to covary with other samples was criteria for sample exclusion. These procedures are described extensively in a previous publication (LaPaglia et al., 2018). Using these criteria, three samples were excluded based on poor sample quality based on lack of covariation in all expressed genes. Expression values were calculated as sFPKM (significant fragments per kilobase of transcript per million mapped reads; MAGIC pipeline), a normalized expression metric that adjusts for several sources of variability including library size, gene length, insert size of the library, and level of genomic contamination. Note that while statistics are performed on raw counts, normalized counts are used by convention in visualizations. Statistics were performed using limma’s voom function (in R) by considering each of the 5 major groups (Male Frontal Cortex, Male Hippocampus, Male Amygdala, Female Hippocampus, Female Amygdala) separately and comparing over time (Control, 1 h, 10 h, Recovery). The section of the script pertaining to this declaration is found in Supplementary Material.

The individual brain regions do not express uniform baseline expression profiles and therefore were analyzed individually. Our goal to identify key transcriptional pathways on a regional basis for further investigation. Note that in this method of analysis we treat each brain region as a separate experiment as there is no reason to expect that all brain regions would be modulated in the same manner.

### Visualization of RNA-Seq Data Using Heatmaps and Scatter Plots

**FIGURE 3:**
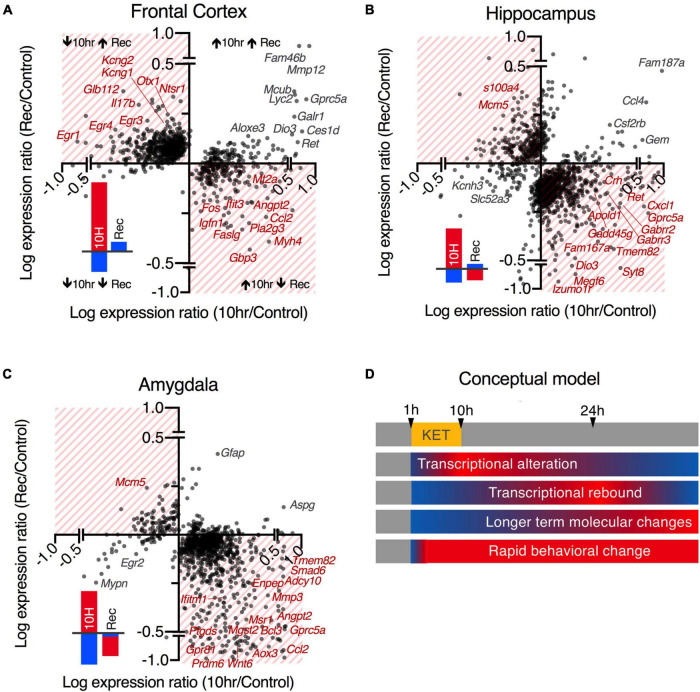
Comparison of 10 and 24 h recovery gene changes. **(A–C)** Expression ratios are compared between the 10 h/control data and the recovery/control. Log-transformed expression ratios of 10 h vs. control (x-axis) and recovery vs. control (y-axis) are shown for all DEGs by brain region. Genes that show rebound transcriptional changes opposite to the direction of regulation at 10 h are highlighted in hatched quadrants (quantified in the red and blue bar graphs in lower left quadrants). Example genes with the highest levels of discordant regulation at 10 h and Rec are labeled in red. Genes with the highest levels of concordant regulation (do not return to baseline) are in the clear quadrants and are labelled in gray. Note that there appears to be a tendency toward increased numbers of genes in the hatched quadrants generally, and that there is more tendency for the genes to move in the opposite direction in the recovery among genes decreasing in the frontal lobe **(A)** or increasing in the hippocampus **(B)** or amygdala **(C)**. **(D)** A conceptual model is shown for the temporal dynamics of the observed transcriptional events. Transcriptional events, which are the primary endpoints of RNA-Seq, have known temporal dynamics with a T_1/2_ of mRNA estimated at ∼2.5–3 h (Singer and Penman, 1972). The 10 h time point was selected because it is about 4 half-lives, implying near total turnover. Return to transcriptional homeostasis after withdrawal of the drug does not necessarily coincide with the end of the ketamine-induced process, as translational processes, protein level, post-translational modifications, network-level, and behavioral alterations may persist. The observations at the 24 h time point also suggest a tendency toward transcriptional overshoot or “rebound” rather than return to homeostasis.

Hierarchical clustering and heatmap visualizations were performed on expression values (sFPKMs) for all differentially expressed genes in R. Data were scaled in R and clustered according to the ward.D2 method (Murtagh and Legendre, 2014) and visualized with the heatmap.2 function. For the plots in Figure 3 showing the degree of rebound, we screened for highly rebounding genes by correlating the expression ratios between control and 10 h and between control and recovery. This yielded a plot with 4 quadrants corresponding to the 2 × 2 set of possible outcomes (increasing vs. decreasing in each comparison). Generally, this showed a trend wherein more points landed in the quadrants corresponding to a rebound effect (i.e., a gene that increases at 10 h and decreases in recovery relative to control). Subsequently, the magnitudes of these effects were estimated by brain region and direction of the difference, as the rebound was generally more prominent for genes moving in the dominant direction in a particular brain region at 10 h (Supplementary Figure 4). At 10 h the rebounding genes were generally decreasing in the frontal cortex and increasing in amygdala and hippocampus. Thus, at the recovery time point, these genes showed an increase in the frontal cortex, and a decrease in amygdala and hippocampus. Small red and blue bar chart icons summarizing these data are located in the in bottom left corners of Figure 3 panels A-C and the rebound quadrants are highlighted by red hatching.

**FIGURE 4:**
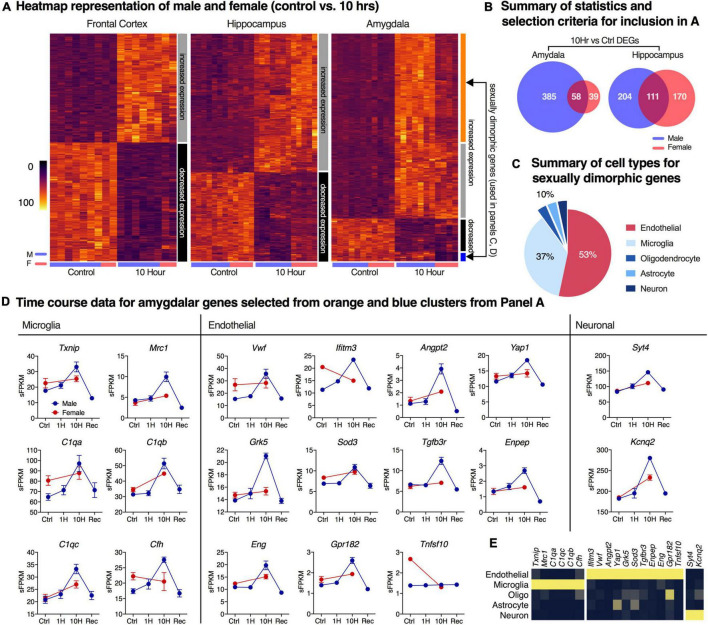
Analysis of sexually dimorphic differentially expressed genes after ketamine infusion. Differentially expressed genes (DEGs) in control vs. 10-h groups in both male and female datasets were identified independently using pairwise comparisons in limma. **(A)** Hierarchical clustering was performed for genes that were differentially expressed at 10 h in at least one sex. The color bar to the right of each heatmap shows the clusters. DEGs show largely concordant induction (gray) or downregulation (black) between sexes in all brain regions. In the amygdala, two additional sexually dimorphic gene clusters were observed (orange, blue). **(B)** Non-concordant amygdaloid genes were categorized by cell type by referring to basal cell expression dataset from a previously published resource (Zhang et al., 2014). Cell type representation in the top 30 most non-concordant genes are shown, demonstrating large endothelial and microglial signatures. **(C,D)** Genes meeting discordance thresholds with high levels of specificity to cell type are shown (red = female, blue = male). **(E)** Expression data from Zhang et al. (2014) for endothelial cells, microglia, oligodendrocytes, astrocytes and neurons are shown for the selected genes in D, showing their high degree of enrichment in their respective cell types according to that dataset. The microglia genes mainly represent the complement cascade. The endothelial genes include a GPCR for the vasodilatory peptide adrenomedullin, a GPCR kinase, as well as genes in the TGFβ family and others consistent with a complex network of endothelial regulation. Error bars show the standard error of the mean.

For the male vs. female comparison heatmaps, control and 10 h data were considered by brain region. Genes significant in either dataset were selected to test for correlation of genes significantly altered in either sex. Because the RNA-Seq analysis was performed to limit type I error (false discovery rate), it is not necessarily informative that the identity of the significantly differential genes is different, as there could be substantial type II error. Therefore, we applied a screen that specifically selects for highly discordant genes (genes which show a trend toward highly divergent expression ratio between sexes). While k-means clustering and non-significant trends are not strong evidence of significant gene changes on a gene-by-gene basis, the purpose of this screen was to identify whether there were generally any trends toward sex differences in the dataset. Male vs. female comparison clustering and heatmap visualization were performed as described above, with the following adjustments. Male and female 10 h expression values (sFPKM) were manually scaled to their respective sex-specific average controls; input data thus reflected normalized expression ratios. The genes selected for most sex-discordancy are shown in Figure 4 (orange and blue clusters) and additional individual plots. These genes were tested for significance by building a secondary model using control and 10 h time point data for male and female rats and testing Sex*Ketamine, but there were no significant genes detected using this analysis.

### Pathway Identification and Analysis

Subsequent analysis was performed to identify Gene Ontology term enrichment using Ingenuity Pathway Analysis (IPA, Qiagen) and the DAVID Bioinformatics Resource (Huang et al., 2009). Outputs from Ingenuity are shown in Supplementary Table 9. The raw outputs from Ingenuity contained several pathways that were of low priority for the study. As an alternative, we selected pathways that highlighted genes that were strongly expressed with the highest fold changes in our dataset (Supplementary Figure 6), and used the existing literature to refine and build gene networks that were relevant to the present study. Gene Ontology term enrichment analysis was performed for males based on the maximum expression ratio between any two time points within the time course, as calculated in Supplementary Figure 3. For females, the expression ratio between control and 10 h was used for Gene Ontology analysis. For heatmaps displaying pathway alterations, the individual gene lists were supplemented with literature review to construct a more comprehensive pathway. The end result was curated to the present dataset relative to the generic pathways from Gene Ontology analysis. Log2 expression ratios for each of the 6 datasets examined (3 brain regions x 2 sexes) were used to create heatmaps of the individual pathways. These pathways also were clustered as described above using the ward.D2 method.

### Evaluation of *Bdnf* Splice Variant Differences Using JunctionSeq

The *Bdnf* transcript is known to play an important role in ketamine actions and to be alternatively spliced into mature transcripts that have differential functional effects and cellular localizations (Baj et al., 2011). In order to assess exon-level differences in gene expression in general, and *Bdnf* in particular, the sequence files were re-aligned using STAR (2.5.0a_alpha), a 2016 Ensembl genome build, and annotations (Rnor_6.0.84) to create standard Binary Alignment Map (BAM) files. Quantification was performed using QoRTs v0.3.18 and JunctionSeq (v3.8; Hartley and Mullikin, 2015). While differential exon statistics were calculated on the whole dataset, we focused on *Bdnf* because we had identified *Bdnf* as one of the most highly differential genes in the dataset both by RNA-Seq and by *in situ* hybridization. Note that BDNF pathway signaling was significant in all three male datasets (Supplementary Table 6) indicating robust modulation of this pathway across brain regions.

### Comparison of the Present Dataset to Single-Cell and Sorted Brain Cell Populations for Estimation of Cellular Origin

In order to estimate the cellular origin of the gene transcriptional responses, two additional datasets from the literature were queried. In the analyses in Figure 4, sexually dimorphic genes were compared to a sequencing database created from purified cell types from mouse cortex (Zhang et al., 2014). This dataset maps the basal expression by cell type in mouse brain, specifically in neuronal and non-neural support cells including oligodendrocytes and microglia. Note that while several subpopulations of oligodendrocytes exist in the original paper, these data were averaged to get a single oligodendrocyte population for simplicity. These methods were derived from a similar strategy used in our report on transcriptional alterations in spinal cord after an experimental surgical incision (Raithel et al., 2018). In the Supplementary material, we also assess the enrichment of the significant genes in a single-cell database of mouse brain (Zeisel et al., 2015; see “Supplementary Material”).

### Multiplex Fluorescent *in situ* Hybridization

Animals were deeply anesthetized with isoflurane for < 3 min and perfused intracardially with cold phosphate buffered saline, followed by 4% paraformaldehyde. The brain was removed from the skull and divided into left and right hemispheres and post-fixed with 4% paraformaldehyde for a minimum of 12 h but not more than 24 h. The frontal lobe was isolated anterior to corpus callosum and marked with dye. A ∼3mm coronal slice containing CA1–CA3 and the amygdala was then cut. All tissue was embedded in paraffin blocks and mounted as 6 μm sections by Histoserv Inc. (Germantown, MD). Sections from the paraffin blocks were taken starting from the caudal aspects of the embedded frontal lobe and the from the rostral face of the hippocampus/amygdala block. Sections from paired control and 10 h animals were mounted on one slide, stained, and scanned concurrently. RNAscope^®^ Multiplex Fluorescent assays v2 (Advanced Cell Diagnostics, Newark, CA) with Tyramide Signal Amplification (Opal*™* Reagent Systems; Perkin Elmer, Waltham MA) was used for multiplex *in situ* hybridization. Staining protocols followed manufacturers’ recommendations. The following Advanced Cell Diagnostics (Newark, CA) RNAscope^®^ Probes were used: Rn-Grin1 (Cat No. 317021), Rn-Grin2C (Cat No. 543951), Rn-Nr4a1 (Cat No. 414571), Rn-Bdnf (Cat No. 317541), Rn-Fos (Cat No. 403591), Rn-Sst (Cat No. 412181). Image capture from the stained sections was performed with a Axio Imager.Z2 slide scanning fluorescence microscope (Zeiss, Oberkochen, Germany) equipped with a 20X/0.8 Plan-Apochromat (Phase-2) non-immersion objective (Zeiss), a high-resolution ORCA-Flash4.0 sCMOS digital camera (Hamamatsu), a 200W X-Cite 200DC broad band lamp source (Excelitas Technologies, Waltham MA) and 5 customized filter sets (Semrock, Rochester NY) optimized to detect the following fluorophores: DAPI, Opal520, Opal570, Opal620, Opal690. Image tiles (600 × 600 μm viewing area) were individually captured at 0.325 micron/pixel spatial resolution, and the tiles seamlessly stitched into whole specimen images using the ZEN 2 image acquisition and analysis software program (Zeiss). Stitched images were overlaid as individual layers to create multicolored merged composites and subsequently analyzed using Fiji version 2.0.0-rc-69/1.52n (Schindelin et al., 2012) to quantify signal intensity and area of puncta. For each stained section, a region of interest (ROI), as determined by anatomical landmarks according to a rat brain atlas (Paxinos and Watson, 2014) was specified and the total number of puncta per intensity measurement was recorded. Puncta counts were then binned per 10 intensity levels (0–255). Each animal served as a statistical unit (one section quantified per animal). For representative images, color thresholds were applied equally to matched control and experimental images to distinguish color balance in Adobe Photoshop (version 20.0.0). To show a signal at low magnification color intensity was substantially enhanced for representative images in some cases. This enhancement was performed uniformly throughout the whole section and was performed identically for controls and ketamine-treated animals. Note that the *Nr4a1* signal in the 620 channel was not quantified in terms of signal intensity due to signal bleed from the 570 channel (*Fos*). However, unique *Nr4a1* puncta (without pixel overlap from the 570 channel) were used to count *Nr4a1* + cells.

### Statistical Analyses

Behavioral data in Figures 2B,C were analyzed in Prism 8 (GraphPad, La Jolla, CA) using a mixed-effects model (thermal), or binomial test (pinprick) with Holm-Šidák corrections. Data are reported as mean ± standard error of the mean (SEM). The pinprick response occurs within 50 ms in controls (Blivis et al., 2017), and was suppressed completely after ketamine. Note that no variance was observed for this measurement. Statistical analysis and differential gene expression determinations were done on raw counts generated by MAGIC, using limma voom (Benjamini-Hochberg adjusted *p* < 0.01; R, Bioconductor; Ritchie et al., 2015). Only the B-H adjusted *p*-values are reported, although nominal *p*-values are available in the Supplementary Tables. Genes were further filtered by expression ratio and absolute expression as follows: the greatest degree of change in expression was determined by establishing the maximum expression ratio among the three major pairwise comparisons in the present study (control vs. 10 h, 10 h vs. recovery, control vs. 1 h). This maximum expression ratio served as the threshold to establish a minimum fold-change based on basal expression values following the formula 2^(1-log(Max sFPKM))*(0.4) + 1. This formula demands a variable change in expression ratio based on sFPKM, with 40% change required to meet threshold at 10 sFPKM. The differentially expressed genes that meet this threshold are screened for both the degree of change and absolute expression (Supplementary Figure 3). For male vs. female comparisons, a separate statistical analysis was performed comparing the control vs 10 h groups within each sex to create a statistic for the males more comparable to the single pairwise comparison (control vs. 10 h) performed in the females. Statistics for differential exon usage of *Bdnf* (Figure 6E) were calculated in JunctionSeq (v3.8) using standard parameters. Technical files used in this analysis are available upon request. Binned puncta counts for control and 10 h animals in Figures 6K,L were analyzed in Prism 8 (GraphPad, La Jolla, CA) using a repeated-measures 2-way ANOVA with Holm-Šidák corrections.

### Identification and Analysis of Genes Significant Across Multiple Datasets

In order to address the most highly significant genes in the dataset we created a filter based on significance (meeting threshold) in three of the five individual experiments in which significance was tested. The list of genes, significance values, and expression data for these genes is found in Supplementary Table 10). Maximum expression ratios (calculated as the maximum change across the time course) for each of the six datasets in the paper were sorted by average expression ratio and plotted in a heatmap (Figure 7A). The top 10 increasing, and top 5 decreasing genes (Figure 7B) with a maximum sFPKM ≥ 5 were selected and sorted by expression ratio. Note that for the decreasing genes this is in ascending order such that the genes decreasing most are first. Three additional categories of genes were selected. Receptors and ion channel genes were selected due to their importance in pharmacology research. Heat shock protein genes were also selected because of their high level of expression.

## Results

### Characterization of the Arousal State During Ketamine Infusion

Chronically catheterized animals were infused with ketamine for 1 or 10 h (Figure 2A), with the infusion dose titrated to obtain a loss of righting reflex and then tested for responses to nociceptive stimulation (see “Materials and Methods”). Ketamine-infused animals showed decreased responsiveness to two types of nociceptive stimuli relative to awake animals, as demonstrated by increased latency to C-fiber selective radiant thermal stimuli, (Figure 2B) as well as the loss of responsiveness to pinprick stimulation, which is largely A-delta-mediated (Figure 2C; Blivis et al., 2017). The consistent blockade of these responses throughout the infusion, as well as the low variance in these measurements confirms stable and efficacious blockade due to ketamine infusion.

### Signatures of Gene Regulation by Brain Region

In addition to the 1 and 10 h infusion groups, a cohort of animals were recovered from ketamine exposure for 24 h after a 10 h infusion (Figure 2A). From the limma analysis of each of the five datasets in the paper: Male Frontal Cortex, Male Hippocampus, Male Amygdala, Female Hippocampus, Female Amygdala, tables of significant genes were constructed summarizing the increasing and decreasing genes in each condition meeting the threshold we established (see “Materials and Methods”). Summary of the number of animals used in the study, as well as the number of significant genes are presented in Table 1. The individual genes comprising this summary table are found in Supplementary Table 5. These genes are also presented in Table 2 (male frontal cortex), Table 3 (male hippocampus), and Table 4 (male amygdala). The equivalent data for the female rats is shown in Supplementary Table 5.

**TABLE 1:** Summary table of RNA-Seq data from six experiments.

	Sample number (*N*)				Number significant genes (*p* < 0.01)	
Number of rats	Ctrl	1 hr	10 hr	Rec	Increasing	Decreasing
Male frontal cortex	7	6	6	6	314	567
Male hippocampus	7	5	7	6	1109	370
Male amygdala	6	6	7	6	992	142
Female frontal cortex	2	–	2	–	–	–
Female hippocampus	5	–	5	–	180	101
Female amygdala	5	–	5	–	175	105

*This table shows the exact number of animals in every experimental group. In the right side of the table, the total number of significant genes are shown for each experiment, broken down by increasing or decreasing genes. Note that each brain region was treated independently. Overlap between significance in different datasets is shown in Supplementary Figure 14 and Supplementary Table 8.*

**TABLE 2:** Male frontal cortex top 20 increasing and decreasing genes.

Gene	Control	1 h	10 h	Recov	10 h/Cont	Adj. P-val
***Top 20 increasing genes, male frontal cortex***						
*Maff*	0.25	1.06	1.65	0.15	4.99	6.98E-06
*Ret*	1.10	1.07	5.14	1.32	4.37	8.89E-05
*Crem*	4.01	7.43	12.56	4.33	3.08	6.98E-06
*S100a8*	0.72	1.33	2.39	0.76	3.03	4.02E-03
*Il6r*	4.55	5.83	13.00	5.39	2.82	6.98E-06
*Acer2*	6.47	8.85	17.84	6.09	2.73	1.97E-05
*Mt2A*	33.73	32.01	89.62	24.49	2.65	1.38E-05
*Fosl2*	4.07	7.26	10.48	4.51	2.53	1.86E-04
*Angpt2*	1.03	1.14	2.72	0.69	2.49	3.94E-05
*Slc4a11*	2.89	4.24	7.27	3.09	2.46	1.18E-03
*S100a9*	1.59	1.47	3.99	1.22	2.42	7.06E-04
*Ankdd1a*	0.47	0.73	1.19	0.62	2.26	2.28E-04
*Nfil3*	1.48	2.36	3.43	1.43	2.23	6.98E-06
*Tex15*	0.63	0.78	1.52	0.79	2.22	7.33E-03
*Hmgcs2*	1.22	1.35	2.76	1.31	2.17	7.73E-03
*Cfap70*	4.05	4.61	8.72	4.92	2.12	3.06E-05
*Rgs16*	5.32	7.27	11.39	5.13	2.12	1.64E-05
*Fosb*	2.75	6.08	5.93	2.78	2.12	7.32E-04
*Mc4r*	0.92	1.35	2.04	1.20	2.10	4.10E-03
*Upp1*	3.20	3.68	6.82	3.41	2.10	6.85E-04
***Top 20 decreasing genes, male frontal cortex***						
*Egr2*	2.92	1.67	0.40	2.86	0.16	3.09E-03
*Egr1*	50.58	33.52	10.36	65.14	0.21	1.64E-05
*F2rl2*	1.08	1.03	0.34	0.93	0.37	8.49E-03
*Tslp*	1.90	1.95	0.70	2.00	0.40	5.46E-03
*Gypc*	2.50	2.72	0.97	2.93	0.41	1.71E-04
*Egr4*	3.36	3.85	1.36	4.97	0.42	5.46E-03
*Tnfaip3*	3.96	4.04	1.67	4.02	0.43	1.86E-04
*Sstr3*	8.15	8.60	3.60	9.37	0.45	6.98E-06
*Prkcdbp*	9.51	10.75	4.29	13.33	0.46	5.71E-05
*Kcnv1*	41.09	37.86	19.00	46.38	0.46	2.92E-06
*Trpm4*	3.20	3.95	1.44	3.73	0.47	1.09E-04
*Dact2*	25.75	24.62	11.99	27.01	0.47	5.78E-05
*Stard8*	12.59	14.22	5.94	13.04	0.48	6.02E-04
*Ptgs2*	13.52	9.62	6.39	13.38	0.48	2.40E-05
*Lrrc17*	1.65	1.89	0.74	1.73	0.48	7.87E-04
*Bace2*	2.67	2.78	1.23	2.69	0.48	3.13E-03
*Kcnh4*	5.19	5.32	2.45	6.07	0.48	1.50E-04
*Lor*	1.34	1.35	0.60	1.70	0.49	1.39E-05
*Lrrc75a*	2.66	3.35	1.27	3.41	0.50	1.50E-03
*Rara*	5.26	6.22	2.57	5.59	0.50	7.08E-04

*These genes were selected by finding significant genes that met threshold (see “Materials and Methods”) and additionally requiring a maximum sFPKM value of 1 within any treatment category. The top 20 genes by expression ratio are shown. Increasing genes are shown at the top (red flame scale) and decreasing genes are shown at the bottom (blue flame scale).*

**TABLE 3:** Male hippocampus top 20 increasing and decreasing genes.

Gene	Control	1 h	10 h	Recov	10 h/Cont	Adj. P-val
***Top 20 increasing genes, male hippocampus***						
*Ccl2*	0.05	0.13	7.38	0.08	49.88	1.2E-08
*Ccl7*	0.07	0.03	4.66	0.03	28.48	2.3E-07
*Maff*	0.22	0.71	5.55	0.25	17.49	2.1E-12
*FAM187A*	0.11	0.38	1.89	0.36	9.27	3.1E-04
*Atf3*	0.06	0.36	1.13	0.05	7.77	2.9E-08
*Lif*	0.08	0.15	1.10	0.07	6.50	9.1E-10
*Socs3*	0.35	0.31	2.69	0.29	6.17	8.5E-11
*Hmox1*	3.74	3.53	22.43	2.99	5.86	2.1E-12
*Angpt2*	0.69	0.89	2.82	0.53	3.68	9.6E-09
*Gem*	1.33	1.42	5.08	1.77	3.61	5.7E-08
*Cd44*	7.42	7.06	25.35	6.27	3.38	1.4E-10
*Acer2*	5.57	6.42	18.28	4.98	3.24	2.7E-08
*Cebpd*	4.48	3.45	14.17	3.96	3.11	1.9E-04
*Aspg*	0.41	0.33	1.47	0.50	3.10	7.0E-05
*Clcf1*	0.70	0.88	2.29	0.60	2.99	1.4E-09
*Mt2A*	41.70	39.94	119.18	35.18	2.85	8.3E-10
*Tnfrsf12a*	2.01	2.17	5.89	1.57	2.85	1.8E-08
*Smad6*	0.29	0.45	1.00	0.26	2.85	6.9E-07
*Hspb1*	3.02	3.55	8.75	2.87	2.84	1.8E-04
*Flnc*	6.33	5.84	17.67	5.23	2.77	7.1E-09
***Top 20 decreasing genes, male hippocampus***						
*Stra6*	1.36	1.20	0.46	1.15	0.39	4.69E-05
*Egr1*	38.35	33.75	15.94	37.13	0.42	2.35E-04
*Slc38a5*	1.43	1.46	0.66	1.19	0.50	2.76E-03
*Lrrc75a*	2.57	2.57	1.30	2.07	0.52	7.85E-07
*Ptgs2*	16.97	16.27	9.13	15.55	0.54	5.74E-06
*Cyp2j10*	3.90	3.46	2.08	3.61	0.54	7.11E-03
*Plekha2*	6.95	6.80	3.73	5.39	0.54	5.50E-04
*Car7*	11.25	10.81	6.13	10.98	0.55	1.94E-06
*Kcnv1*	13.28	13.67	7.34	15.05	0.56	3.41E-06
*Slc52a3*	1.22	1.16	0.65	0.88	0.56	9.80E-03
*Tnc*	5.63	5.92	3.13	5.55	0.56	2.00E-05
*Nxpe1*	1.58	1.52	0.86	1.79	0.57	5.75E-05
*Tie1*	6.00	5.25	3.43	4.54	0.58	8.43E-06
*Serpinb6b*	0.90	0.55	0.48	1.09	0.58	3.66E-04
*Gdpd2*	1.86	1.16	1.06	1.67	0.59	2.32E-03
*Kcnh4*	2.33	2.43	1.36	2.50	0.60	1.66E-04
*Rbm3*	79.57	88.54	47.99	101.12	0.60	2.63E-06
*Slc26a7*	2.10	2.03	1.24	2.09	0.61	1.06E-03
*Prom1*	10.16	10.73	6.14	8.53	0.61	2.49E-04
*Car4*	15.59	15.16	9.46	15.04	0.61	1.21E-03

*These genes were selected by finding significant genes that met threshold (see “Materials and Methods”) and additionally requiring a maximum sFPKM value of 1 within any treatment category. The top 20 genes by expression ratio are shown. Increasing genes are shown at the top (red flame scale) and decreasing genes are shown at the bottom (blue flame scale).*

**TABLE 4:** Male amygdala top 20 increasing and decreasing genes.

Gene	Control	1 h	10 h	Recov	10 h/Cont	Adj. P-val
***Top 20 increasing genes, male amygdala***						
*Maff*	0.31	0.92	2.56	0.41	6.52	1.5E-05
*S100a9*	0.47	1.13	2.82	0.42	5.09	3.2E-03
*Inmt*	0.30	0.66	1.69	0.13	4.47	1.2E-03
*Acer2*	4.57	7.30	20.37	4.04	4.39	6.6E-07
*Aspg*	0.27	0.25	1.45	0.50	4.25	1.0E-03
*Ccl2*	0.17	0.30	1.04	0.03	4.24	5.5E-05
*Crem*	2.80	6.27	12.01	2.90	4.18	2.9E-06
*Ttr*	4.24	3.79	17.78	3.56	4.12	7.8E-03
*Tmco4*	0.62	0.64	2.74	0.53	3.96	1.1E-06
*S100a8*	0.35	0.38	1.66	0.46	3.88	3.9E-03
*Spta1*	0.35	0.26	1.57	0.30	3.69	1.2E-04
*Mt2A*	19.48	19.51	64.71	20.03	3.31	9.9E-06
*Angpt2*	1.13	1.29	3.92	0.52	3.28	7.4E-06
*Inhba*	2.11	4.28	7.12	2.02	3.26	6.8E-03
*Ret*	7.39	5.52	24.01	7.87	3.22	4.2E-04
*Smad6*	0.29	0.46	1.12	0.25	3.09	2.7E-05
*Tmem82*	0.27	0.23	1.00	0.24	2.95	1.8E-04
*Insrr*	0.32	0.50	1.11	0.03	2.90	9.1E-04
*Hmox1*	3.08	4.16	9.14	3.81	2.90	2.2E-05
*Lox*	0.77	0.74	2.37	0.36	2.85	5.8E-04
***Top 20 decreasing genes, male amygdala***						
*Stra6*	1.18	1.09	0.41	0.80	0.40	9.90E-03
*Egr1*	50.04	35.29	20.78	37.52	0.42	6.49E-04
*Klra1*	1.23	1.18	0.51	1.17	0.46	3.46E-03
*Car7*	5.65	7.48	3.11	5.63	0.56	3.58E-05
*Kcnv1*	21.29	22.45	12.38	23.66	0.58	1.08E-06
*Egr2*	2.65	2.68	1.55	2.18	0.60	2.84E-04
*Lsp1*	1.68	1.39	0.98	2.16	0.61	6.66E-04
*Cd180*	2.35	1.87	1.41	2.81	0.62	1.94E-03
*Trim54*	20.42	21.96	12.63	19.98	0.62	5.26E-04
*Rnd1*	21.32	24.33	13.57	22.64	0.64	1.15E-04
*Ciart*	6.41	6.38	4.09	7.78	0.64	8.70E-05
*Zdhhc22*	24.73	26.46	15.88	30.08	0.64	1.93E-05
*Oas1b*	1.68	1.60	1.05	2.31	0.64	3.04E-04
*Gpr12*	4.06	4.10	2.58	3.82	0.64	2.84E-04
*Fam180b*	3.71	3.29	2.36	3.98	0.64	1.08E-06
*Tmem266*	4.02	4.33	2.56	4.43	0.65	3.76E-06
*Pkp2*	3.73	3.96	2.38	3.43	0.65	3.60E-03
*Rbm3*	103.53	100.52	67.54	95.15	0.65	2.15E-03
*Lurap1*	9.16	9.16	6.01	10.58	0.66	3.73E-07
*Mas1*	4.81	6.20	3.16	4.98	0.66	2.36E-05

*These genes were selected by finding significant genes that met threshold (see “Materials and Methods”) and additionally requiring a maximum sFPKM value of 1 within any treatment category. The top 20 genes by expression ratio are shown. Increasing genes are shown at the top (red flame scale) and decreasing genes are shown at the bottom (blue flame scale).*

Heat maps were constructed to enable the data to be assessed in a summary fashion. In the hippocampus and amygdala, the majority of differentially expressed genes were upregulated (75 and 87% respectively), whereas 64% of genes in the frontal cortex were downregulated (Figure 2D). For each region, the heat map is divided into genes whose expression is increased or decreased according to the direction of transcriptional change at the 10 h time point. Note how strongly the 10 h time point is differentiated from the controls, the 1 h and the 24 h recovery points (Figure 2E). This differential degree is evident in all three brain regions. Analyses of specific pathways activated are presented in the “Functional pathways” section below.

### Rebound Gene Regulation Seen in All Brain Regions

At 24 h after cessation of the 10 h infusion, many genes either exhibited full recovery or rebound changes in the opposite direction (e.g., genes upregulated at 10 h are more likely to be *downregulated* at recovery and vice versa; Figures 3A–C). This effect was most pronounced in genes changing in the same direction as the major pattern of transcriptional regulation within each brain region at 10 h. In the frontal cortex, the rebound effect was more prominent among the genes that decreased at 10 h, whereas in the amygdala and hippocampus, where the majority of gene changes observed were increases, the rebound decrease was more pronounced among these increasing genes (Figures 3A–C, Supplementary Figure 4). The largest rebound effects were observed in the frontal cortex and amygdala, where the rebound effect was ∼50% of the main effect among strongly regulated genes (Supplementary Figure 4). A scatter plot showing all genes with the log expression ratio at 10 h relative to the log expression ratio at recovery shows a general tendency for rebound (Figures 3A–C). Graphical summaries of the results in Supplementary Figure 4 are also shown next to each scatter plot, summarizing the effect for the most highly differential genes. While it is difficult to perform statistics on the presence of rebound for an individual gene, the general trend is significant for the dataset as a whole (see statistical testing in Supplementary Figure 4). Individual genes showing rebounding trends are highlighted in Supplementary Figure 5. Analyses of specific pathways activated are presented in the “Functional pathways” section below.

### Sex Differences in Transcriptional Response to Ketamine

In order to determine whether sex differences occurred in response to ketamine, the 10 h time point was examined in both male and female rats. This time point was selected because it showed the strongest gene changes. In all three brain regions, patterns of gene up- and downregulation are generally correlated between sexes (Figure 4A). The male animals showed a larger number of differentially expressed genes. In hippocampus, there were 315 differentially expressed genes in the males, and 281 in the females. In the amygdala, the males showed 443 differentially expressed genes while the females showed just 97 (Figure 4B). In the amygdala, two subsets of ketamine-regulated genes show sexual dimorphisms (orange and blue clusters), following patterns of either male-specific upregulation or female-specific downregulation. However, these clusters represent non-significant trends, and were generally of small magnitude. Genes identified as sexually dimorphic were further characterized based on cell-type specific expression profiling from an RNA-Seq database of purified cell types from mouse cortex (Zhang et al., 2014; Zhang et al., 2016). Genes with the highest degrees of discordance between sexes show predominantly vascular or immune signatures denoting endothelial and microglial cell populations, respectively (Figures 4C–E). A small number of neuron-enriched genes such as synaptotagmin 4 (*Syt4*) and the potassium channel *Kcnq2* were also identified, but are not genes highly specific for neurons (Zhang et al., 2004), and, while showing a more accentuated up-regulation in males, are not among the most highly discordant genes (Figure 4D). However, it is notable that synaptic changes involving the paralog synaptotagmin 1 have been observed in response to ketamine (Muller et al., 2013). Similarly, Kcnq2 has been reported in pyramidal cells as a regulator of intrinsic excitability (Niday et al., 2017), which would support modulation of neuronal function associated with this transcriptional change. Also note that in the dataset at large, the majority of the genes appear to be of neuronal origin (Supplementary Figure 13), as significant genes are enriched in neurons vs. other cell types. Note that in both sexes, drug administration was titrated to the same plane of behaviorally defined anesthesia (loss of righting and withdrawal reflexes), and the relatively minor differences in transcriptional alterations between males and females may in part be due to dosing and pharmacokinetic differences. However, in our hands alteration of dosing between males and females was necessary to maintain stable physiologic parameters.

### Functional Pathways

**FIGURE 5:**
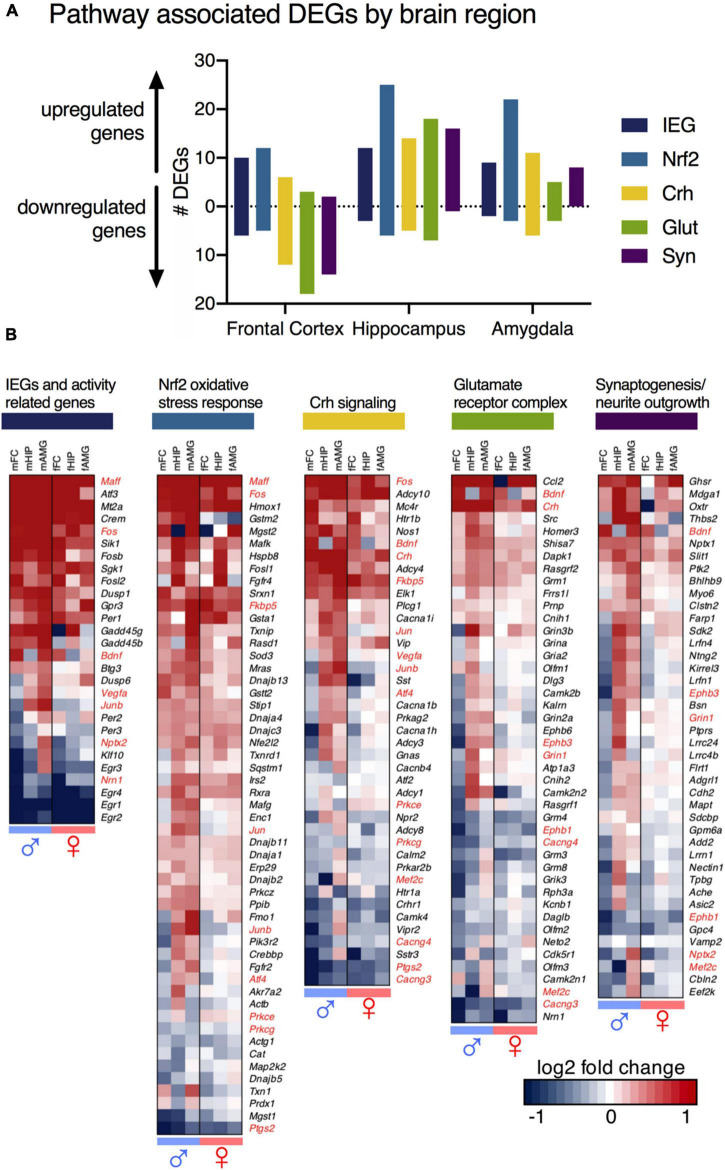
Pathway analysis of differentially expressed genes (DEGs). DEGs that met threshold from male and female animals were analyzed for canonical pathway enrichment with Ingenuity Pathway Analysis, and Gene Ontology (GO) term enrichment DAVID Bioinformatics Resources. Four key molecular pathways are highlighted – Corticotropin releasing hormone (Crh) signaling, Nrf2 (Nuclear factor-like 2) - mediated oxidative stress response (Nrf2), Glutamate receptor complex regulation (Glut), and Synaptogenesis (Syn). **(A)** Total number of genes in identified pathways, by brain region. All but one molecular pathway (Nrf2) show patterns of predominant upregulation in the hippocampus and amygdala, and downregulation in the frontal cortex. The hippocampus shows the most robust change in the Nrf2 pathway. **(B)** Changes in each pathway, across sexes and brain regions. Genes significant and meeting threshold in at least one brain region in at least one sex for each pathway are shown. Subclasses of upregulated and downregulated genes are seen. Regional differences from male animals are mirrored as non-significant trends in female animals. Genes mapped to multiple pathways are highlighted in red.

To identify important pathways for neuronal control and communication, differentially expressed genes from male and female animals were analyzed for canonical pathway enrichment with Ingenuity Pathway Analysis, and Gene Ontology (GO) term enrichment in DAVID Bioinformatics Resources and supplemented by literature searches (Figure 5). Five key molecular pathways were identified (Figure 5A). A brief description of each of these pathways is found below. These pathways were constructed initially based on the raw outputs from Ingenuity (Supplementary Table 9), and were referenced against the existing literature as well as using the scatter plots in Supplementary Figure 6 to select for significant genes that were highly expressed with a strong fold change. Note that absence of evidence is not evidence of absence with respect to pathway alterations, and that this is a selection of the most prominent changes. The full list of significant genes is found in the Supplementary data files. We additionally performed gene ontology analysis in Enrichr on the three male datasets (Supplementary Table 6). However, we believe our curated lists to be easier to comprehend than the outputs of that package due to the multifunctionality of most genes identified. Note that genes may have several functions, and that in some cases were identified as being associated with multiple of the below pathways (and indeed in many other functions in biology). Also note that in some cases, while significant, the individual gene changes are quite small, which drives our decision to focus on pathways rather than individual candidates. In the examples below we give detailed expression ratio information and *p*-values for specific genes/brain regions. For succinctness we provide the data for one dataset, but most of these genes are differential in multiple datasets.

1. IEGs and activity-related genes. These are genes that have been previously identified to increase with neuronal activity and are involved in activity-transcription coupling. These genes are transcriptional correlates of brain activation. Some of the genes included in this category include the well-studied activity related genes *Fos*, *Junb*, and the Early Growth Response genes, *Egr1*, *Egr2*, *Egr3*, and *Egr4*. *Egr1* in particular is strongly down-regulated in several datasets (decreasing in the male frontal cortex, 10 h/control expression ratio = 0.21, *p* = 1.64 × 10^–5^). Note also that the Fos family genes *Fosb* (increasing in male frontal cortex, expression ratio = 2.12 *p* = 7.32 × 10^–4^) and *Fosl2* (increasing in male frontal cortex, expression ratio = *2.*53 *p* = 1.86 × 10^–4^) are among the most strongly regulated genes in the male frontal cortex as well.
2. Nrf2 (nuclear factor, erythroid 2 like 2) mediated oxidative stress response. This is another activity-coupled gene transcription pathway that is activated downstream of the activation identified in pathway 1 (activity-related genes). This oxidative stress response is thought to be related to a glutamate surge occurring with neuronal activity and has been associated with NMDA receptor antagonism in previous studies. This gene category includes genes such as Heme Oxygenase 1 (*Hmox1;* increasing in male hippocampus, expression ratio 10 h/control = 7.29, *p* = 2.07 × 10^–12^), Sulfiredoxin 1 (*Srxn1*; increasing in male frontal cortex 10 h/control expression ratio = 1.72; *p* = 6.98 × 10^–6^), and thioredoxin interacting protein (*Txnip;* increasing in male amygdala, expression ratio of 10 h/control = 1.86, *p* = 0.0000549). Note that Nrf2 itself (*Nfe2l2*) was not significant.
3. Corticotropin releasing hormone (Crh) signaling. This is a stress hormone signaling pathway that has been described previously in relation to the clinical actions of ketamine. This category of genes included *Crh* itself (increasing in male hippocampus, expression ratio 10 h/control = 1.98, *p* = 2.09 × 10^–5^) as well as Fk506 binding protein 5 (*Fkbp5*, increasing in male amygdala, expression ratio 10 h/control = 1.86, *p* = 0.00021).
4. Glutamate receptor complex regulation. This is a collection of genes related to regulation of glutamate receptors and receptor trafficking pathways. Notable in this pathway were Ephrin type-B receptor 6 (*Ephb6*, decreasing in male frontal cortex, expression ratio = 0.77, *p* = 0.00017) and Ephrin type-B receptor 3 (*Ephb3*, decreasing in male frontal cortex, expression ratio = 0.62, *p* = 0.00226). These are receptor tyrosine kinase transmembrane glycoproteins that have roles in axon guidance and dendritic spine development. This pathway also included several glutamate receptor subunits with small but significant changes such as *Grin1*, *Gria2*, and the metabotropic glutamate receptors *Grm3* and *Grm8*.
5. Synaptic regulation. Similar to what is described in pathway 4, these genes modulate synaptic proteins to maintain or modulate synapses. Notable among this category was neuronal pentraxin 2 (*Nptx2*, decreasing in male frontal cortex, expression ratio = 0.77, *p* = 0.0085), a gene involved in excitatory synapse formation and AMPA glutamate receptor clustering. Another notable gene in this pathway was the oxytocin receptor (*Oxtr*, expression ratio between 10 h and recovery = 2.75, *p* = 0.00235)

In general, pathway-associated gene groups were more downregulated in the frontal cortex (Figure 5A), and upregulated in the hippocampus and amygdala, with the exception of Nrf2-mediated oxidative stress responses (shown in light blue) and activity-related genes (dark blue) which were induced in all three brain regions. Activity-related genes emerged from the data because they are genes that generally correlated with neuronal activity, and as such are a molecular correlates of brain activity. The Nrf2 pathway is also the most highly represented pathway in aggregate, which is seen in the heatmaps showing all members ascertained to be part of the pathway (Figure 3B). Scatter plots were constructed to show the relationship between pathway, expression level and expression ratio (Supplementary Figure 6). The pathway analysis was also useful in classifying the rebounding genes. Of the genes with the highest rebound effect in the frontal cortex, for example, several are identified in the immediate early gene cluster (Supplementary Figures 5, 7). Notably, a degree of interaction between the five major functional pathways is reported in the present paper, with proteins such as the NMDA receptor being predicted as central nodes of the interaction network (Supplementary Figure 8).

Based on the suggestion that neurogenesis could be involved in ketamine’s actions, we examined the dataset for clusters of genes associated with other functions including a panel of hippocampal genes related to neurogenesis (Supplementary Table 7). However, with several exceptions, most of the genes related to neurogenesis were not significantly altered with either 1 or 10 h infusions of ketamine using either transcriptomic results or by protein staining in cortical and hippocampal tissue sections (Supplementary Figures 9, 10).

Ingenuity pathway analysis (IPA, Qiagen) was also performed on the most sexually dimorphic genes, identifying several pathways enriched in the male animals relative to females including the Nrf2 pathway (Supplementary Figure 11A). However, the male and female datasets showed good correlation in general, and the magnitude of the sex differences in the Nrf2 pathway was small (Supplementary Figures 11B–E).

### Regional Induction of Plasticity-Related Transcripts in Medial Cortex and *Bdnf* Splice Variant Analysis

**FIGURE 6:**
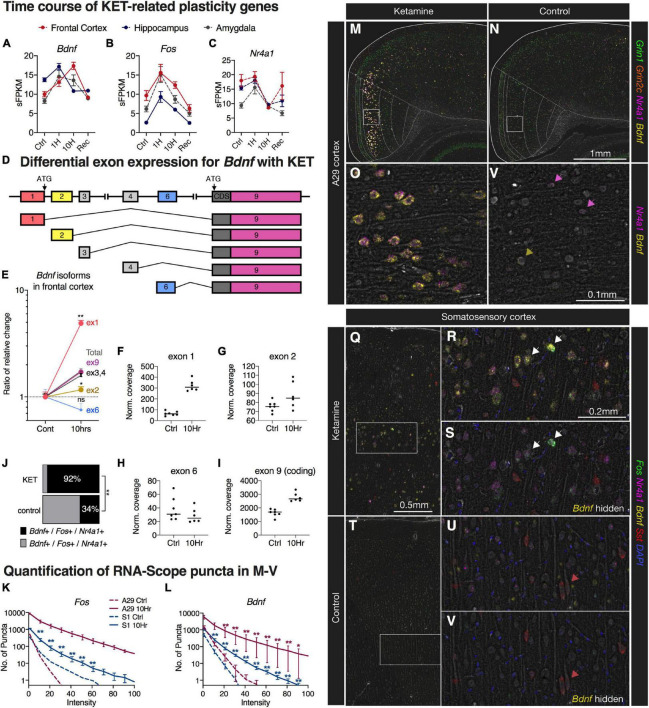
Multiplex fluorescent *in situ* hybridization, quantification, and splice analysis of selected ketamine-induced cortical neuroplasticity genes. **(A–C)** Expression level of *Fos*, *Bdnf*, and *Nr4a1* were measured, each showing differing patterns of expression over time. Transcripts for *Bdnf* showed notably different expression patterns between brain regions, while the other two genes showed consistent effects in these three brain regions. **(D)** The *Bdnf* gene contains several exons, which all splice to the coding exon 9. (**E–I)** Exon-level expression was assessed using JunctionSeq showing a significant increase in exon 1 variants relative to the total, whereas exon 2 was not induced by ketamine. **(J–V)** Using *in situ* hybridization, we investigated the effects of ketamine on specific transcripts. **(J)** After ketamine, significantly more *Bdnf* + /*Fos* + cells also contained *Nr4a1* + puncta. **(K,L)** Induction of both *Fos* and *Bdnf* transcripts was observed in cortical pyramidal neurons after 10 h of ketamine administration, with larger magnitudes of effect size observed in A29 cortex. This was manifested as an increase in intensity of the staining. **(M,N)** A representative image is shown for A29 retrosplenial cortex, with a medial induction pattern. **(O,P)** In the magnified images, strong staining is noted in large pyramidal cells with weaker staining and less co-expression observed in controls (colored arrows). **(Q–S)** A similar effect was observed in deeper layers of somatosensory cortex, although less pronounced. **(T–V)** Control somatosensory cortex showed faint staining for *Fos*/*Nr4a1*/*Bdnf*, while somatostatin staining (red arrows) remained relatively constant. **(M–V)** Brightness was enhanced for visibility at low power for this display. Statistical tests used: **(E)** JunctionSeq statistics; **(J)** Chi-square analysis; **(K,L)** Repeated measures two-way ANOVA on log-transformed values; *, *p* < 0.05; **, *p* < 0.01. For all panels, error bars represent the standard error of the mean. Brightness in these images was enhanced for visibility at low power.

Three indicators of molecular plasticity were examined in greater depth using neuroanatomical *in situ* hybridization. Two genes, *Fos* and *Bdnf* were selected because of their high degree of significance, high degree of change, and prominence in the pathway analysis (Figure 5). We examined nuclear receptor subfamily 4 group A member 1 (*Nr4a1*), a transcription factor in the steroid-thyroid hormone-retinoid receptor superfamily, because of its established role in plastic events (Chen et al., 2014; Jeanneteau et al., 2018). Expression level of *Fos*, *Bdnf*, and *Nr4a1*, as measured by RNA-Seq, showed differing expression patterns of over time for each gene. Within the frontal cortex, *Fos* and *Bdnf* show elevated expression with *Fos* exhibiting the highest levels at 1 h, and with *Bdnf* being increased most strongly at 10 h (Figures 6A,B). While *Bdnf* showed notably different temporal expression patterns between brain regions, the other two genes showed consistent patterns of expression in all three brain regions (Figures 6A–C). One potential explanation for the complexity of the *Bdnf* induction signature is the simultaneous regulation of multiple splice variants serving different functions (Baj et al., 2011). The *Bdnf* gene contains several non-coding 5’ exons (Figure 6D), which all splice to the coding exon, exon 9. Exon-level quantification of expression was assessed using JunctionSeq (Hartley and Mullikin, 2016), which measures relative exon expression within a gene product. For *Bdnf* transcripts, in the frontal cortex at the 10 h time point, exon 9 closely matches the overall increased expression of the transcript since it is a component of all variants. Exon 1 was significantly increased at the exon level, indicating stronger regulation of the exon 1-containing pool of *Bdnf* transcripts. Conversely, exon 2-containing variants were not as strongly regulated as the overall transcript, and exon 6-containing variants showed a non-significant downward trend (Figures 6E–I).

Using multiplex *in situ* hybridization, the expression of these three genes was assessed in cortical coronal sections with prominent staining occurring in medial cortex and, more laterally, throughout the deeper layers of somatosensory cortex. The strongest upregulation occurred in the A29 region (retrosplenial cortex). Co-expression of *Nr4a1* with *Fos* and *Bdnf* was significantly increased by ketamine such that the majority of *Fos* + /*Bdnf* + neurons were also *Nr4a1* + (92%, Figure 6J). The overall *in situ* hybridization signal for *Fos* and *Bdnf* was quantified by examining the distribution of puncta across intensity values, showing significantly increased frequency of more intensely fluorescent puncta in 10 h ketamine sections relative to control (Figures 6K,L). Quantification of induction of *Nr4a1, Fos, and Bdnf* in the A29 cortical region is shown in Figures 6M–P. A similar effect was also observed in somatosensory cortex (Figures 6Q–S) and coronal sections through frontal cortex (Supplementary Figure 12). The *N* is shown in tabular format for these experiments in Supplementary Figure 12. The somatostatin (*Sst*) expressing neurons that were labeled in somatosensory cortex showed no obvious difference between control and ketamine sections (Figures 6Q–V).

### Analysis of Genes Significant in Multiple Brain Regions

We noted some overlap in the significance of genes in different brain regions (Supplementary Figure 14, Supplementary Table 8). While each brain region may behave differently in response to ketamine, we performed an analysis to search for genes that were highly significant (met threshold) in 3 or more comparisons (the three male brain regions, and two female brain regions in which statistics were performed). Unexpectedly, these genes (with the sole exception of *Ccl2*, shown as part of the glutamate receptor complex pathway in Figure 5) always changed in the same direction in every dataset. Notably, the one dataset in which *Ccl2* diverges has very low expression and only *N* = 2 animals (female frontal cortex, statistics not performed). This indicates that among the most robustly significant genes, there is often a similar trend throughout brain regions and across sexes. To focus on these genes, we further selected the top genes by expression ratio, with *Ccl2* being the highest fold change overall. However, *Ccl2* is mostly expressed in the hippocampus (7.38 sFPKM in males and 16.64 in females) whereas it is marginally detected in other regions (≤ 1.04 sFPKM). Several other genes identified as part of the pathways in Figure 5 were also detected including *Maff*, *Hmox1*, and *Crem*. Among the decreasing genes, *Egr1* (part of the activity-regulated genes in Figure 5) was the most strongly decreasing gene, showing strong expression in all three brain regions of both sexes. Heat shock proteins were also selected due to 5 heat shock related proteins being identified in this analysis. All of these genes exhibited very strong expression, with the HSP90 family member Hsp90ab1 detected at over 1000 sFPKM in the hippocampus. Ion channels and receptor genes were highlighted due to their relevance to pharmacology research. We selected receptor genes ≥ 1 sFPKM in any dataset, which is less stringent than the requirement of 5 sFPKM for the other datasets due to the fact that receptor genes are often lowly expressed in RNA-Seq studies (Sapio et al., 2016; Sapio et al., 2018; Sapio et al., 2021). The interleukin 6 receptor (*Il6r*) was the most strongly regulated receptor gene and is one of the top 10 increasing genes overall with robust expression in all three brain regions (sFPKM ∼10–20). The FK-506 binding protein 5 (*Fkbp5*) was also among the most strongly regulated receptor associated genes with strong expression in all brain regions (∼20–40 sFPKM). The three ion channel-related genes in this analysis were all highly expressed, with two decreasing (*Cacng3* and *Kcnv1*) and one increasing (*Kcnq2*). Due to the prevalence of immune-like signaling genes, we also highlighted the highly-expressed microglial marker genes *C1qa* and *C1qb* (∼30–100 sFPKM) which increased across the datasets (increase of ∼30–60% in male brain regions).

## Discussion

The essential findings of the present study reveal patterns of transcriptional networks and diverse molecular regulatory mechanisms over time that are potentially informative to ketamine’s mechanisms of action *in vivo*. Importantly, while the dosing was high compared to clinical use (Carboni et al., 2021), the transcriptional effects are modest and largely resolve by 24 h implying limited long term transcriptional impact of ketamine administration. This report represents a holistic approach to identifying genes and pathways involved in ketamine action. At its core this is a basic science investigation in a rat model, using a dose that is outside what is generally used clinically. However, the identified changes accentuate the molecular modifications that are likely operational with therapeutic doses used for treatment of chronic pain and depression. Clinically, longer administrations and higher dosing has led to higher magnitude and longer duration of effects (Borsook, 2009), and we expect that to some extent the current investigation likely exaggerates the findings that would be observed at a lower dose. However, some of these basic pharmacological questions are still being worked out. We demonstrate that the high magnitude changes from tissue collected acutely at 1 and 10 h precede and predict those occurring after a 24 h recovery. This points to the importance of assessing both acute and longer-term transcriptional changes to understand intravenous ketamine action. The utility of this approach can be expanded to ketamine analogs and metabolites, which are also being investigated to optimize and clarify therapeutic interventions for pain and depression, both of which are frequently comorbid conditions. Beyond this, the longitudinal transcriptomic investigational approach is useful to achieve better molecular understanding of anesthetic drugs or perioperative drug combinations where the molecular etiology of some therapeutic effects is unknown.

This report examines the effects of ketamine over time, by sex, and in three major brain regions. The combination of short and sustained administrations, in conjunction with the comprehensive nature of next-generation sequencing, enabled examination of systems-level consequences of ketamine treatment. We observed overlapping, as well as differential, transcriptional modulation of activity-related genes between brain regions. In all three areas, the more accentuated effects obtained with a 10 h infusion are consistent with the known kinetics of mRNA turnover in the brain. The hippocampus and amygdala showed mainly induction of activity-coupled genes, whereas the frontal cortex had signatures of both increasing and decreasing activity. The key signaling pathways, including the Nrf2 pathway, were uniformly elevated across the three brain regions. At the cortical level, this supports the hypothesis that ketamine induces a generalized glutamate surge by inhibition of interneurons and consequent activation of pyramidal cells throughout neocortex. The involvement of *Bdnf-*expressing neurons is also a prominent feature in cortex and a detailed examination of exon utilization in the transcriptomic dataset shows differential splicing consistent with expression of *Bdnf* splice variants involved in proximal dendritic remodeling and plastic events (Baj et al., 2011; Leal et al., 2014). Thus, several effector pathways for intra- and intercellular communication are defined, some broad, and others such as *Bdnf* splicing, very specific.

Animal studies of ketamine are frequently carried out using subanesthetic doses with tissue harvested hours to weeks after drug delivery. These later time points are relevant to subsequent behavioral outcomes on the recovered animal, but do not necessarily capture the acute, direct effects of ketamine on transcription. The present study builds upon previous studies of effects of ketamine by extending these investigations to transcriptional changes occurring *during* the ketamine infusion and subsequently at the 24 h recovery. At 10 h of ketamine infusion, the gene regulatory events are substantially greater than those observed either 1 h into the infusion, or 24 h after recovery. We suggest based on this evidence that later time points, which have been the focus of many previous studies (Ficek et al., 2016; Bagot et al., 2017; Orozco-Solis et al., 2017; Jacobson et al., 2019) are most likely preceded by acute transcriptional events that are challenging to measure without using long-term infusion paradigms due to the nature of mRNA turnover kinetics. That is, an acute response, potentially of larger magnitude may have preceded and driven the effect that was reported at later time points. An interesting discovery stemming from the time course design was that after recovery for 24 h, the gene changes reverse in many cases, showing up to 50% of the 10 h effect size, yet in the *opposite* direction (Figures 3A–C) suggestive of oscillatory gene regulation patterns over time. It is also very critical to address the fact that previous studies using doses and time points meant to mimic the clinical situation identified comparatively fewer significant gene changes, and changes of a smaller magnitude (Bagot et al., 2017). Together with our study, which includes a 24 h recovery time point, we infer that the most relevant timing in terms of impact on gene expression is while ketamine is still on board, and that these changes become smaller over time. Taken together with the findings of Bagot et al. (2017), this suggests a dynamic where rapid early events found in the present study, may persist or have rebound effects at the time points and/or doses examined in that study.

Unlike other anesthetics, ketamine has been shown to increase indicators of metabolic and neuronal activity in areas such as the thalamus (Langsjo et al., 2005), and frontal and anterior cingulate cortices (Crosby et al., 1982; Furth et al., 2017). High frequency cortical gamma oscillations in particular have been reported in response to ketamine (Lazarewicz et al., 2010; Furth et al., 2017; Nugent et al., 2019b), and are one of several physiological correlates indicating perturbation of thalamocortical integration processes. NMDA receptor antagonism or hypofunction has also been associated with global surges of glutamate across the frontal cortex, (McGirr et al., 2017) as well as thalamo-cortical delta bursting (Zhang et al., 2009; Zhang et al., 2012). The observation that ketamine acts as an anesthetic, and also paradoxically causes cortical activation is consistent with our transcriptomic findings. In the frontal cortex, the majority of genes decrease (64%), but the genes exhibiting increased expression, in general, have a greater magnitude in absolute terms than the decreasing genes (Figures 2, 3). This is suggestive of simultaneous activation and suppression of gene programs in cortex, e.g., some cell types may be strongly activated (Figure 6), while other populations may be inhibited. In a deep brain stimulation study in the subgenual cingulate for depression, stimulation of Brodmann area 25 normalized hyperactive metabolic activity, leading to an improvement in the clinical outcome (depression; Mayberg et al., 2005; Carboni et al., 2021). It is plausible that the decreasing activation signature in the cortical areas is a molecular correlate of such an action, and that both excitatory and inhibitory mechanisms of ketamine’s clinical actions could be simultaneously engaged. NMDAR hypofunction has also been theorized to have a reciprocal link to network disinhibition and an oxidative stress response as seen by induction of the *Nrf2* pathway in response to ketamine infusion (shown in Figure 5; Hardingham and Do, 2016; Kadriu et al., 2019). These acute events may also transition to long-term neuroplasticity through activation of glutamate receptor complexes and modulatory proteins, or modulation of regulatory proteins such as ephrins and pentraxins, which may regulate synaptogenesis and/or longer term plastic changes (Figure 5B).

Sex differences in ketamine response have been raised as an important consideration for molecular studies (Carrier and Kabbaj, 2013; Saland et al., 2017). While sex was seen to affect the number of significant genes reported by the statistical analysis, these programs are designed to pick the highest confidence changes and to reduce false positives. In a correlational analysis, the overall directionality of most genes was highly similar between males and females. The conservative conclusion from this set of observations is that ketamine affects males and females similarly. In the amygdala, we identified two gene clusters that were discordant, contributed from vascular endothelial, and glial genes that were resistant to the effects of ketamine in females. This vascular gene modulatory effect, which was non-significantly trending toward more prominent regulation in males, is consistent with reports that ketamine-induced antidepressant plastic effects are also associated with vascularization that accompanies synaptogenesis (Markham and Greenough, 2004; Ardalan et al., 2017), and with the requirement for vascular endothelial growth factor (VEGF) signaling in ketamine-mediated antidepression (Deyama et al., 2019). In the males, the A isoform of VEGF (*Vegfa*) was elevated in the amygdala. In general, the main finding of this analysis was that male and female rats are affected similarly, and that there were no significant differences identified. However, this is also a complex statistical paradigm. Potentially, the trend toward an amygdalar population of non-neural cells that responds more strongly in males than females could be of interest in future studies if it can be reproduced and studied further. Note that the dosing was titrated to effect, and that males and females required different doses (see “Materials and Methods”).

An interesting feature of the frontal cortex is that we observe overall indications of transcriptional repression, but simultaneously strong activation of select genes involved in neuronal activity-transcription coupling, and synaptic plasticity. The decreasing genes in the frontal cortex specifically have more of an indication of neuronal origin when mapped against markers of neuronal populations from single-cell datasets in the mouse (Supplementary Figure 13; Zeisel et al., 2015). The observed upregulation of markers such as *Fos* was associated with activation of certain subclasses of neurons, particularly pyramidal neurons in the medial and frontal cortical areas (Figures 6M–P). *In situ* hybridization showed that the most apparent activation was in layer V pyramidal cells of retrosplenial cortex (A29). This area is associated with the integration of memory and sensation in rodents, as well as emotional associations of such processes in humans. Retrosplenial cortex forms a key component of the default mode network (DMN) in humans, which is perhaps consistent with studies showing ketamine-induced normalization of aberrant DMN connectivity in depressed patients (Nugent et al., 2019a). This connection is speculative at this time, but something that may be possible to expand upon in future studies. That is, there is a certain degree of limitation to the rat model with regard to the conservation of function in areas such as A29, but it is nevertheless noteworthy that the identified gene changes in this area indicate an activation. In this region of medial cortex, the neurons in which *Fos* is induced also show induction of *Nr4a1* and *Bdnf* suggesting modification of synapse number associated with plastic remodeling in response to ketamine-induced activity (Chen et al., 2014; Haile et al., 2014; Leal et al., 2014; Jeanneteau et al., 2018). Activity-induced *Nr4a1* in particular has been shown to regulate distribution and number of excitatory synapses on pyramidal cells (Chen et al., 2014). Synthesis of BDNF and synapse formation downstream of NMDAR blockade by ketamine is necessary for the antidepressant activity of these compounds (Li et al., 2010; Autry et al., 2011; Duman et al., 2016). These modulations are thought to sustain the normalization of dysfunctional prefrontal cortical circuits by NMDAR antagonists which underlie long-term effects of ketamine (Moda-Sava et al., 2019). Beyond this, *Bdnf* gene regulation is isoform specific, with induction of exon 1-containing *Bdnf* isoforms being more pronounced, with comparatively less regulation of exon 2 and exon 6-containing isoforms. This is consistent with regulation of plastic events at proximal dendrites being more prominent than those at distal dendrites (Baj et al., 2011). These exons (exons 1 and 4) which are more strongly regulated in response to ketamine also appear to be methylated in psychiatric conditions, and this exon-specific methylation status is reported to normalize in response to psychotherapy and symptom improvement (Perroud et al., 2013), suggesting a biological relevance to these isoforms in modulating symptoms such as depression and chronic pain.

**FIGURE 7:**
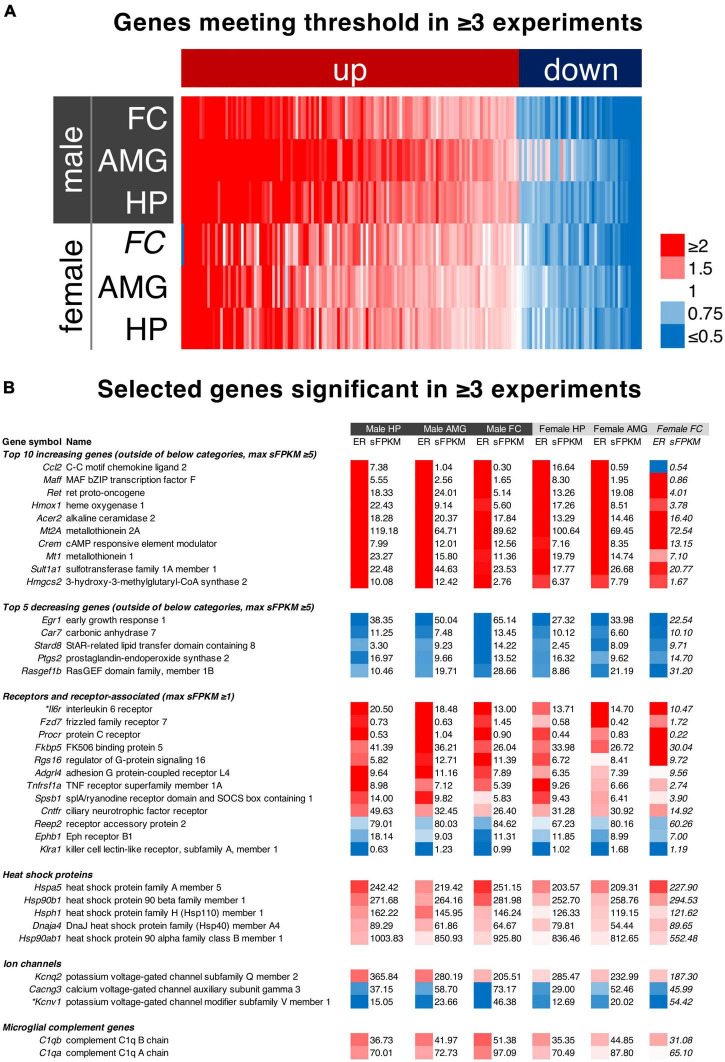
Analysis of genes significant in at least three datasets. In order to examine the most highly significant genes across all brain regions and across sex, we selected genes with a maximum sFPKM ≥ 1 that met the significance threshold in at least three of the five datasets in which statistics were performed (177 genes total). **(A)** These genes were plotted as a heatmap sorted by average expression ratio across the six datasets (the female frontal cortex was included, *N* = 2 rats, statistics not performed). This resulted in a heatmap in which all genes were regulated uniformly across the six experiments, indicating a consistent effect in these three brain regions. **(B)** To focus on individual genes in this analysis, the top increasing and decreasing individual genes are highlighted, showing expression ratio (flame scale) and sFPKM values (average in all samples in the experiment shown). Additionally, groups of genes corresponding to receptor/receptor associated genes, heat shock proteins, ion channels, and microglial complement genes are shown to highlight highly expressed, consistently regulated genes important for pharmacology and neuroscience research.

Another intriguing finding of the present study is that genes that were significant in at least three datasets uniformly changed in the same direction (Figure 7A) suggesting that the most robust gene signature across brain regions is regulated in the same direction regardless of brain structure. This includes some genes which are related to neuronal activity such as the transcription factor *Crem*, which increases, and the transcription factor *Egr1* which decreases. Additionally, two genes involved in lipid synthesis Alkaline ceramidase 2 (*Acer2*) and Prostaglandin-endoperoxide synthase 2 (*Ptgs2*, protein also known as cyclo-oxygenase 2, COX-2) were among the most prominent genes across datasets (Figure 7), potentially implicating lipid processing downstream of anesthetic actions of ketamine. The decrease in *Ptgs2* is consistent with the previous finding that neuronal COX-2 is regulated by spontaneous neuronal activity, and is NMDAR-dependent (Hewett et al., 2016), as it decreases in expression with the infusion of intravenous NMDAR antagonist. The Interleukin 6 receptor (*Il6r*) was the most highly differential receptor gene in our analysis in Figure 7, and has diverse roles in neuronal signaling, and regulation of neuronal plasticity (Gruol, 2015), in addition to other roles in immune signaling. Activation of interleukin 6 signaling has previously been shown in cell culture as a mechanism of ketamine action on neurons (Behrens et al., 2008). The gene encoding Monocyte chemoattractant protein-1/C-C Motif Chemokine Ligand 2 (*Ccl2*) was the overall strongest gene in the analysis in Figure 7, largely driven by its very strong induction in the hippocampus (average sFPKM = 7.4; ER = 49.9, male hippocampus). This protein is known to signal to microglial cells, where it stimulates migration and proliferation (Hinojosa et al., 2011). Notably, the highly expressed microglial marker genes *C1qa* and *C1qb* are also consistently induced, potentially due to microglial proliferation. The C1q complement signaling pathway has been previously identified in RNA-Seq studies in paradigms associated with microglial activation/proliferation (Raithel et al., 2018; Sapio et al., 2021), and this signaling pathway has also been proposed as a mechanism for synaptic remodeling (Stevens et al., 2007).

The long duration and high dose of ketamine used reveals networks of gene regulation induced by the presence of the drug, especially given that tissue sampling was performed immediately after the 1 and 10 h time points. Strengths of the study relate to sample size (*N* = 6–7 animals per time) and our assessment of gene changes over time (4 time points), by sex and in three brain regions. At the same time, a potential limitation of this paradigm is that the dose was at the high end of the clinical use spectrum since we titrated to a plane of anesthesia that blocked thermal nociception. Our reductionistic approach did not use adjuvant medications such as xylazine or midazolam and such combinations can produce their own set of unique actions. Additionally, some of the gene changes may be associated with hallucinatory actions that occur with high doses of ketamine and integrating such alterations of complex cortical circuits (e.g., Figure 6) is an area requiring further investigation. The overall gene changes were less evident at the 1 and 24 h recovery time points suggesting that studies employing either a lower dose or longer-term recovery intervals of ketamine using transcriptional endpoints would be challenging. Protein assays or proteomic analyses may be revealing at longer times. For example, we observed sustained elevations of Fos and Fra-2 protein levels in a spinal dorsal horn laminae the duration of which far exceeded the initial mRNA elevation (Sapio et al., 2021). In this regard the IEGs and synaptic proteins identified in the present study may provide specific targets for examination at the protein and neuronal structural levels.

Ketamine is a unique dissociative anesthetic with interesting clinical utilities. When used in the perioperative period, it can help reduce postoperative pain and opioid requirements. Notably, ketamine is often co-administered with midazolam in clinical care, or with other drugs such as methadone (Kharasch and Clark, 2021; Murphy et al., 2021). In animal studies, ketamine is generally co-administered with xylazine for anesthesia. These combinatorial pharmacological elements can be systematically considered in future mechanistic studies. For example, comparing ketamine to other similar drugs (Ficek et al., 2016), as well as to clinically useful drug combinations could refine the unique mechanistic consequences of ketamine therapy. This could help understand critical barriers, such as improving the clinical profile, which is limited by undesirable psychomimetic side effects, particularly if used as a high-dose monotherapy or in combinations to reduce post-operative opioid use (Kharasch and Clark, 2021). Further understanding of the molecular mechanisms of action on the central and peripheral nervous system could be leveraged to refine the drug action. Presumably, there is a shared dynamic gene signature induced by all anesthetics, including ketamine, most likely involving wide scale transcriptional suppression, which was observed in all three brain regions examined. We also observed transcriptional *activation* that was prominent in the hippocampus and the amygdala. Such paradoxical activation is atypical of anesthetic agents and could be a molecular correlate of ketamine’s unique drug actions. This approach can be expanded to investigate classical anesthetics which in turn would further delineate the contrasts between ketamine’s canonical and non-canonical molecular actions. This knowledge may ultimately prove useful for developing improved ketamine-like anesthetics.

## Data Availability Statement

The datasets generated for this study can be found in the Sequence Read Archive (https://www.ncbi.nlm.nih.gov/bioproject/PRJNA607336).

## Ethics Statement

The animal study was reviewed and approved by the Institutional Animal Care and Use Committee of the National Institutes of Health, Clinical Center.

## Author Contributions

JK, MS, FV, MI, and AM: conceptualization. JK, MS, AL, and DM: investigation. MS, JK, and DM: visualization. MS and JK: formal analysis. JK, MS, AL, FV, and DM: methodology. MS, JK, and MI: drafting the manuscript. MS, MI, JK, WM, AL, CZ, and AM: editing the manuscript. MS, MI, and AM: supervision. DM, CZ, MI, and AM: funding acquisition. All authors contributed to the article and approved the submitted version.

## Conflict of Interest

CZ is listed as a co-inventor on a patent for the use of ketamine in major depression and suicidal ideation. CZ is listed as co-inventor on a patent for the use of (2R,6R)-hydroxynorketamine, (S)-dehydronorketamine, and other stereo-isomeric dehydro and hydroxylated metabolites of (R,S)-ketamine metabolites in the treatment of depression and neuropathic pain; and as co-inventor on a patent application for the use of (2R,6R)-hydroxynorketamine and (2S,6S)-hydroxynorketamine in the treatment of depression, anxiety, anhedonia, suicidal ideation, and posttraumatic stress disorders. He has assigned his patent rights to the US government but will share a percentage of any royalties that may be received by the government. The remaining authors declare that the research was conducted in the absence of any commercial or financial relationships that could be construed as a potential conflict of interest.

## Publisher’s Note

All claims expressed in this article are solely those of the authors and do not necessarily represent those of their affiliated organizations, or those of the publisher, the editors and the reviewers. Any product that may be evaluated in this article, or claim that may be made by its manufacturer, is not guaranteed or endorsed by the publisher.
