# Supplementary material for: Transcriptional Activation, Deactivation and Rebound Patterns in Cortex, Hippocampus and Amygdala in Response to Ketamine Infusion in Rats
Source: Front Mol Neurosci. 2022 May 30;15:892345. doi: 10.3389/fnmol.2022.892345 (PMC9190438; doi:10.3389/fnmol.2022.892345)
Supplement: Supplementary file 9 [file Data_Sheet_1.docx]

Supplementary Material

Transcriptional activation, deactivation and rebound patterns in cortex, hippocampus and amygdala in response to ketamine infusion in rat

**Jenny J. Kim^1*^ M.D., Matthew R. Sapio^1*^ Ph.D., Fernando A. Vazquez^1^ M.D., Dragan Maric^2^ Ph.D., Amelia J. Loydpierson^1^ B.S., Wenting Ma^1^ B.S., Carlos A. Zarate Jr.^3^ M.D., Michael J. Iadarola**^1^ Ph.D., Andrew J. Mannes1 M.D.^1^**

1. Department of Perioperative Medicine, Clinical Center, National Institutes of Health, Bethesda, MD 20892

2. Flow and Imaging Cytometry Core Facility, National Institute of Neurological Disorders and Stroke, National Institutes of Health, Bethesda, MD 20892

3. Experimental Therapeutics and Pathophysiology Branch, National Institute of Mental Health, National Institutes of Health, and Department of Health and Human Services, Bethesda, MD 20892*****

**Supplementary Data: Large Tables**

**Supplementary Table 1.** Expression data (sFPKM) and statistics (limma) for the male animals.

**Supplementary Table 2.** Raw count data for the male animals.

**Supplementary Table 3.** Expression data (sFPKM) and statistics (limma) for the female animals.

**Supplementary Table 4.** Raw count data for the female animals.

**Supplementary Table 5.** Top 20 genes by expression ratio (10 hrs/Control) for each RNA-Seq Dataset.

**Supplementary Table 6.** Enrichr analysis for signficant genes in the dataset.

**Supplementary Table 7.** Genes from neurogenesis pathway shown in in Supplementary Figure 9.

**Supplementary Table 8.** Genes in the Venn Diagram in Supplementary Figure 14.

**Supplementary Table 9.** Raw outputs from Ingenuity Pathway Analysis used to create gene groups.

**Supplementary Table 10.** Expression data for full gene list used to construct Figure 7.

.

## Supplementary Figures


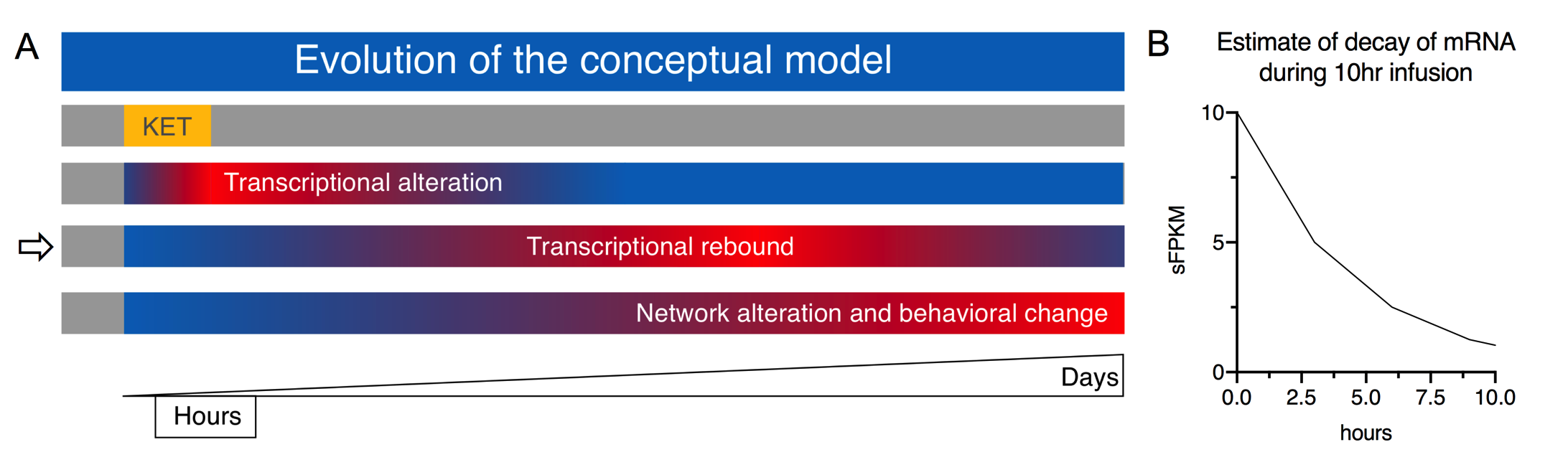


**Supplementary Figure 1. Conceptual model for experimental design. A.** In determining the molecular and pharmacological parameters of the study, we have constructed a model of the experimental design. Transcriptional events, which are the primary endpoints of RNA-Seq, have known temporal dynamics (Singer and Penman, 1973). **B.** Particularly, as shown in panel B, the T_1/2_ of mRNA is about 2.5-3 hours (Singer and Penman, 1972), suggesting that about 10 hours is required to reach near-total turnover of mRNAs. In general, transcription is often rapid and transient, and would be driven most strongly in the presence of the drug. Given these considerations, we selected time points at 1 hour and 10 hours to capture gene changes in response to intravenous ketamine. Namely, in these time points, we capture the transcriptional events driven in the presence of the drug. Once, the drug is withdrawn, transcription will begin to return to homeostasis. Return to transcriptional homeostasis does not necessarily coincide with the end of the ketamine-induced process, as protein level, translational processes, behavioral and network-level alterations may persist. In the present report, we also describe an intermediate phenomenon that we term “rebound,” (indicated by arrow) where transcription overshoots on its return to homeostasis, instead of going smoothly back to baseline. Persistent network-level changes would represent effector processes that behaviorally translate the antecedent transcriptomic alterations.

**
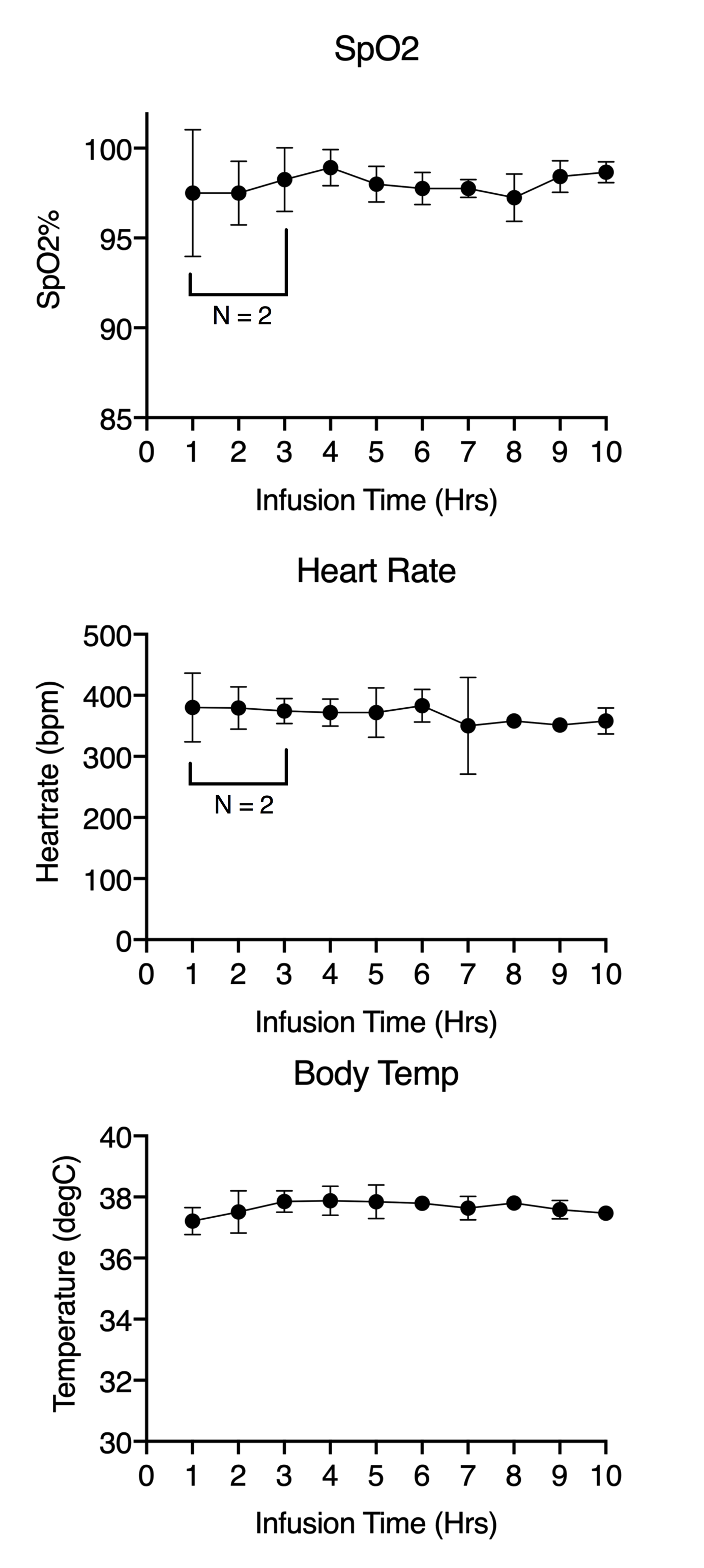
Supplementary Figure 2.** **Hemodynamic status of anesthetized animals.** Pulse oximetry, heart rate, and rectal temperature were measured every 15 minutes in a sentinel animal per infusion group (N=3 animals in each graph). In general, none of these parameters were altered by ketamine. Points represent hourly averages of measurements from 15-minute intervals. Data are reported as mean +/- standard error of the mean. SpO2% and heartrate data were observed but not collected for one sentinel animal for the first three hours due to recording software failures.

**
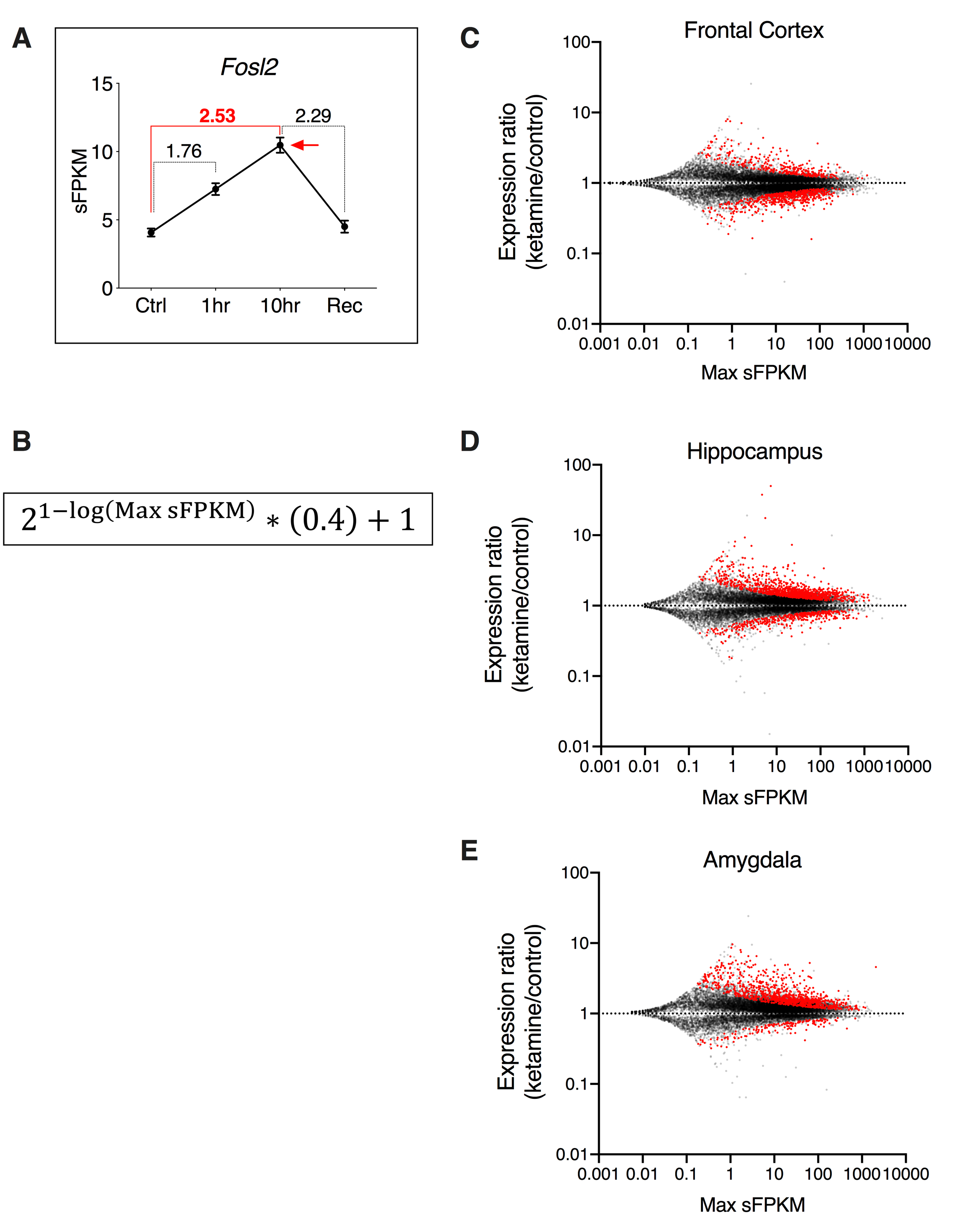
**

**Supplementary Figure 3.** **A.** Example calculation of maximum expression ratio determinations in male animals. In all cases throughout the paper expression ratio was calculated with a small number (0.1) added to both the numerator and the denominator which limits the tendency of expression ratios involving small denominators to trend towards infinity. This also creates the contour in panels C-E because using this formula there is a maximum possible expression ratio within each scatter plot (i.e. the maximum sFPKM per x-value divided by 0.1 for increasing genes). Maximum expression ratios were used in subsequent filtering steps to prioritize genes based on a single numeric value. **B.** Formula used to filter significant genes, which demands a variable expression ratio dependent on sFPKM. In order to meet threshold, a significant gene must have an expression ratio of 40% at 10 sFPKM. A higher expression ratio is demanded of lower expressed genes, and a lower expression ratio is demanded of higher expressed genes, creating the contour between red and black dots in C-E. **C, D, E**. Scatter plots of maximum sFPKM and expression ratio values for each gene by brain region in male animals. These plots are ordered by maximum sFPKM values on the x-axis to show the relationship between expression ratio and expression. In general, higher expressed genes show a lower expression ratio, as expected. Thresholded significant genes (as described in B) are shown in red, while all other genes are show in black. These plots show the overall distribution of significantly differential genes per brain region. The gene lists resulting from this filtering process are used throughout the manuscript.


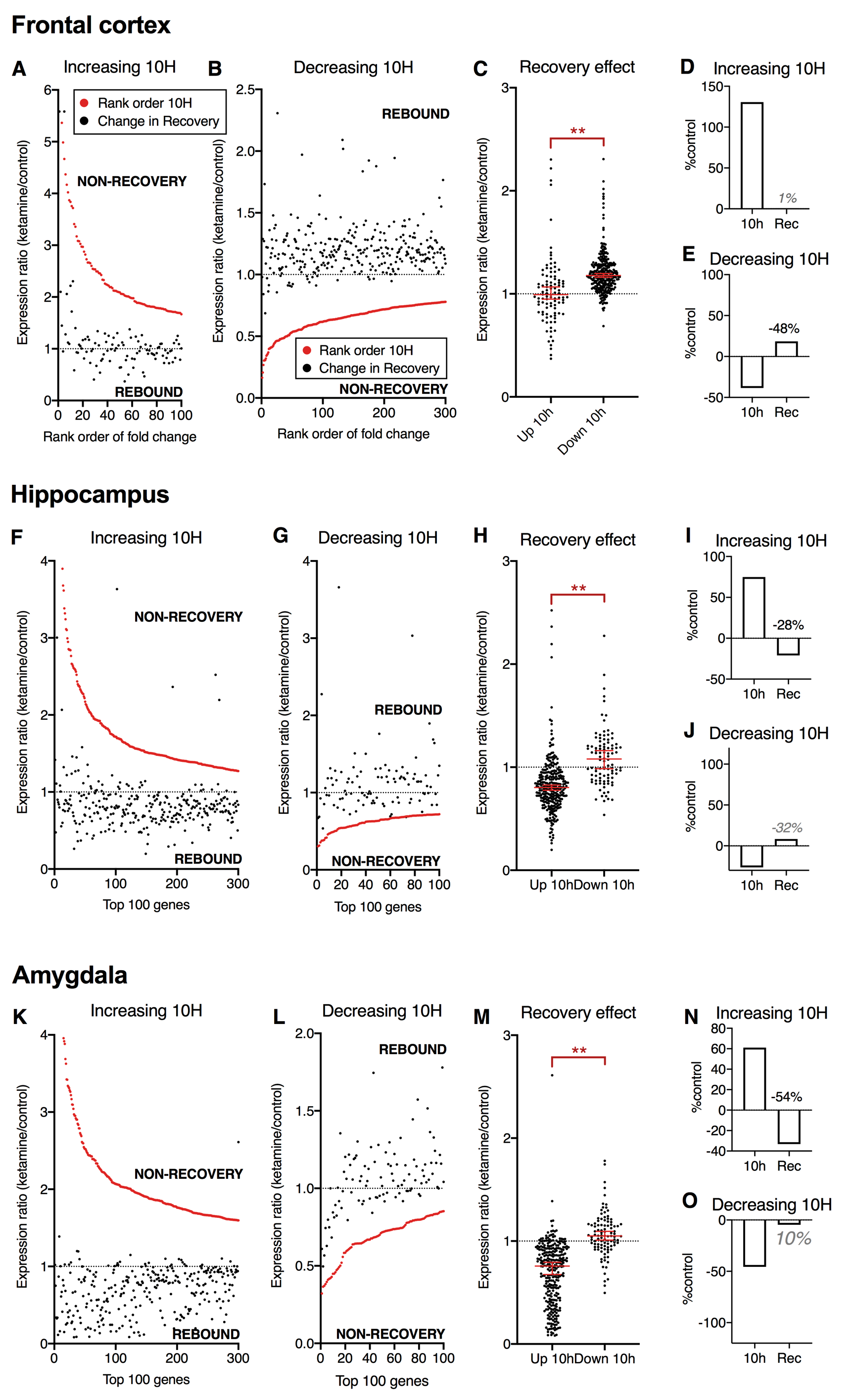


**Supplementary Figure 4. Quantification of rebound effects in the recovery relative to the 10hr gene changes. A-E.** **Frontal Cortex.** Quantification of the frontal cortex gene changes at the recovery time point. **A.** The genes that were increasing at 10hr were selected and plotted in rank order of their 10hr expression ratio (red dots), alongside the gene changes at recovery (black dots). Genes below the dotted line at 1 (no change) are rebounding, whereas those above it are trending towards non-recovery. In the case of this non-recovery, this indicates that the gene has not yet recovered by 24hr and is still trending in the same direction in the recovery as it was at 10hr. The 100 top genes are shown. **B.** An identical plot as in A was constructed for the genes decreasing at 10hr. The 300 top genes are shown (note that this is greater because the number of genes decreasing in the frontal cortex is greater than the number increasing.) The rebounding and non-recovery designations are inverted in **B** relative to **A** to account for directionality of the 10hr change. The same dots for the recovery in **A** and **B** are plotted side by side showing a trend towards increasing in recovery for those genes decreasing at 10hr. The magnitudes of these changes are shown in **D** and **E** with the degree of rebound shown above the recovery bar plot. In D, there is no evidence of rebound or non-recovery on average (1%, labeled with gray text), whereas in **E**, the rebound effect is ~48% the magnitude of the 10hr effect, and in the opposite direction among the top 300 genes by expression ratio (referred to as -48%). **F-H**. Identical plots are shown for the hippocampus. **I.** Among the increasing genes there was an approximately 28% rebound downwards among increasing genes, whereas the decreasing rebound was much smaller in magnitude (although approximately the same in terms of percentage (-32%, labeled with gray text). **K-M.** Amygdala plots. **N.** The rebound among increasing genes in the amygdala was 54% decreasing. **O.** The decreasing amygdala genes showed no evidence of rebound and showed a mild trend towards non-recovery on average. **D, E, I, J, N, O** were used to construct summary pictograms in Figure 1. Statistical significance was assessed in panels C, H and M using a Wilcoxon test. **, p < 0.01.


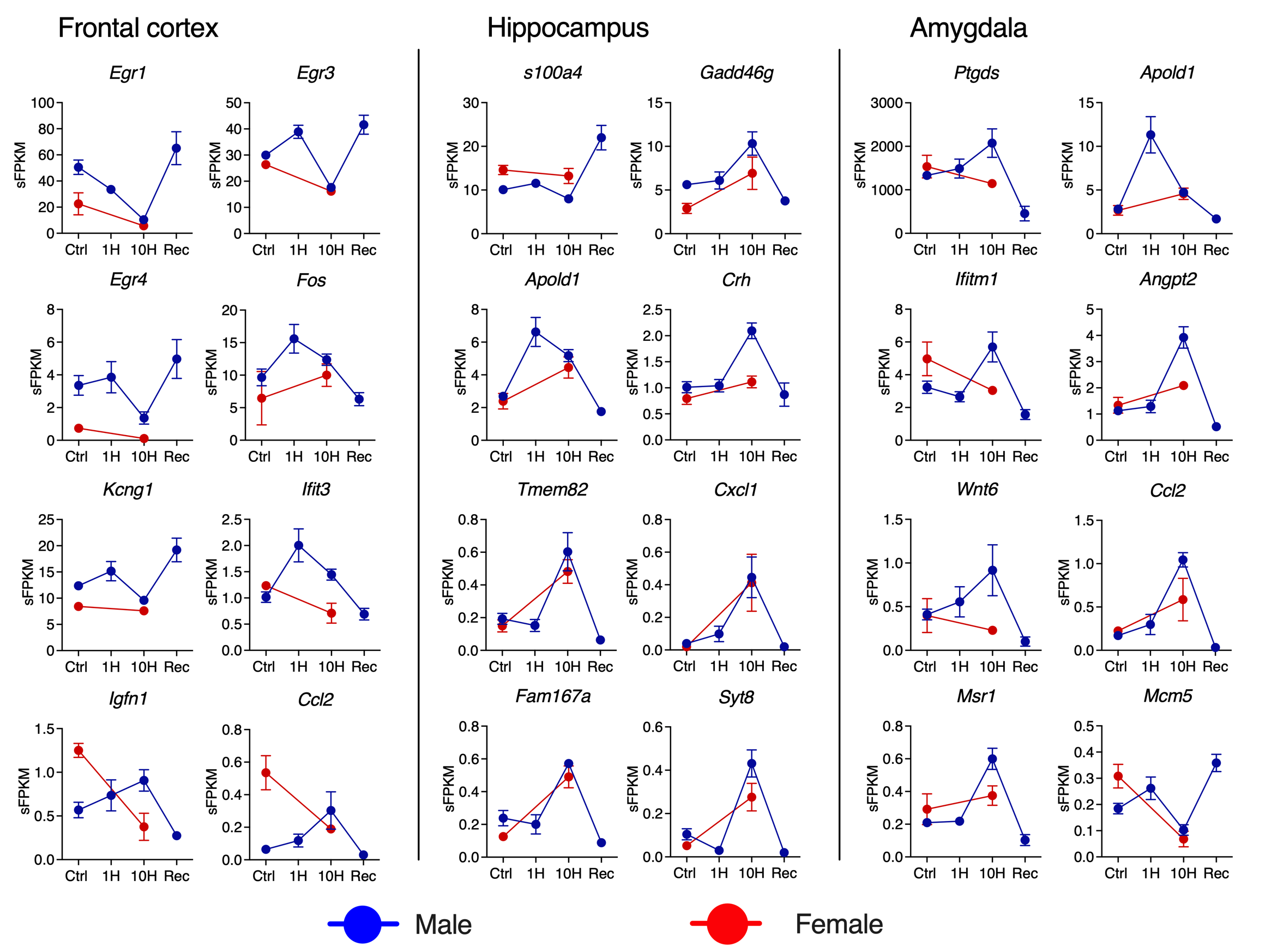


**Supplementary Figure 5. Example genes displaying trends towards a rebound effect at 24hr relative to 10hr and control.** Male and female datapoints are indicated as follows: Male in blue; female in red. Note that females were examined at control and 10 hrs time points only. For any individual gene, the rebound effect is small enough so as to be difficult to observe. We have not performed statistics specifically on the control vs. recovery time point within this manuscript, nor would we expect the majority of these genes to be significant at this time point if pairwise statistics were performed in that fashion. It is only in the aggregate that the “rebound” effect at 24hr becomes apparent. Nonetheless, examples of genes with a trend towards rebound are shown as examples of what the trend looks like. In the simplest cases, such as with *Egr1* there is a mild overshoot phenomenon where the transcriptional level appears to return to a level slightly above where it started. Such a change would most likely be impossible to see reproducibly in a staining experiment such as with *in situ* hybridization because the magnitude is small (much smaller than the main effect at 10hr). In other cases, such as with *Kcng1*, a more complex pattern emerges where there is a possible trend towards increase at 1hr in addition to a trend towards rebound. These individual graphs display why this phenomenon would be difficult to examine on a gene-by-gene basis as the changes are in general minor and highlights the advantage of the RNA-Seq methodology, which measures all transcripts simultaneously. One exception is that of *Ptgds* in the amygdala, which displays a strong decrease at the recovery time point. Note that in the frontal cortex, several genes (*Egr1*, *Egr3*, *Egr4*, and *Fos* especially) are discussed with respect to rebound effects in the context of pathway analysis (Supplementary Figure 6).


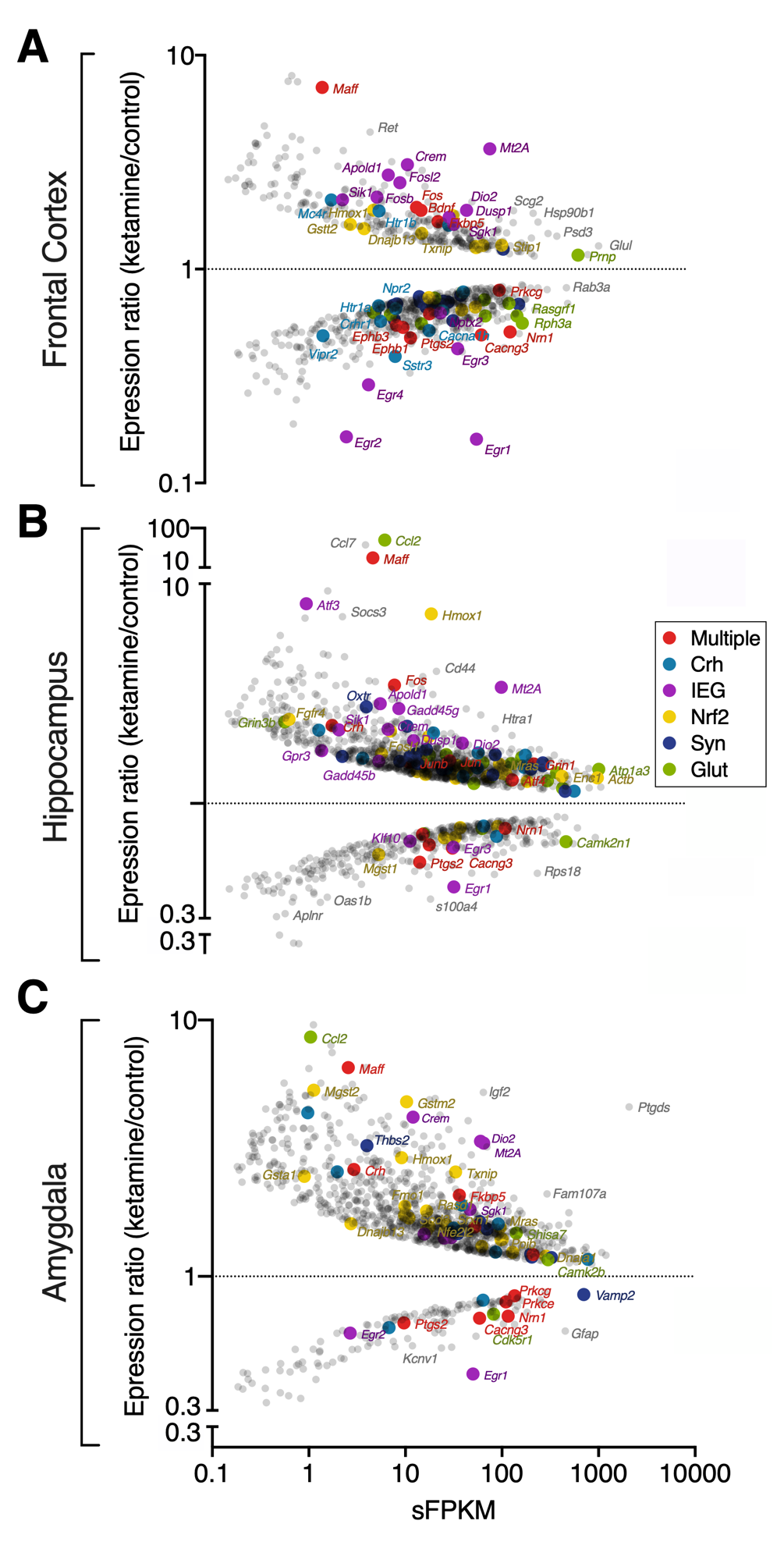
**Supplementary Figure 6. Scatter plots showing expression (sFPKM) and expression ratio of differentially expressed genes colored by associated pathway.** All differentially expressed genes were extracted from the dataset and plotted such that the estimated expression level (sFPKM) is shown on the X-axis, and maximum expression ratio (calculated as shown in supplementary figure 2) is shown on the Y-axis. Data are shown for **A.** frontal cortex, **B.** Hippocampus and **C.** Amygdala. The identity of key genes is highlighted based on expression level and expression ratio. Many of these genes that are highly expressed and/or highly differential are constituents of the pathways shown in Figure 3, showing that these pathways are comprised of some of the more highly expressed highly differential genes. Genes are colored by pathway (light blue, Crh; purple, IEG; yellow, Nrf2; dark blue, synaptic; green, glutamate) with genes associated with multiple of these pathways shown in red. Unassigned genes are shown in gray.

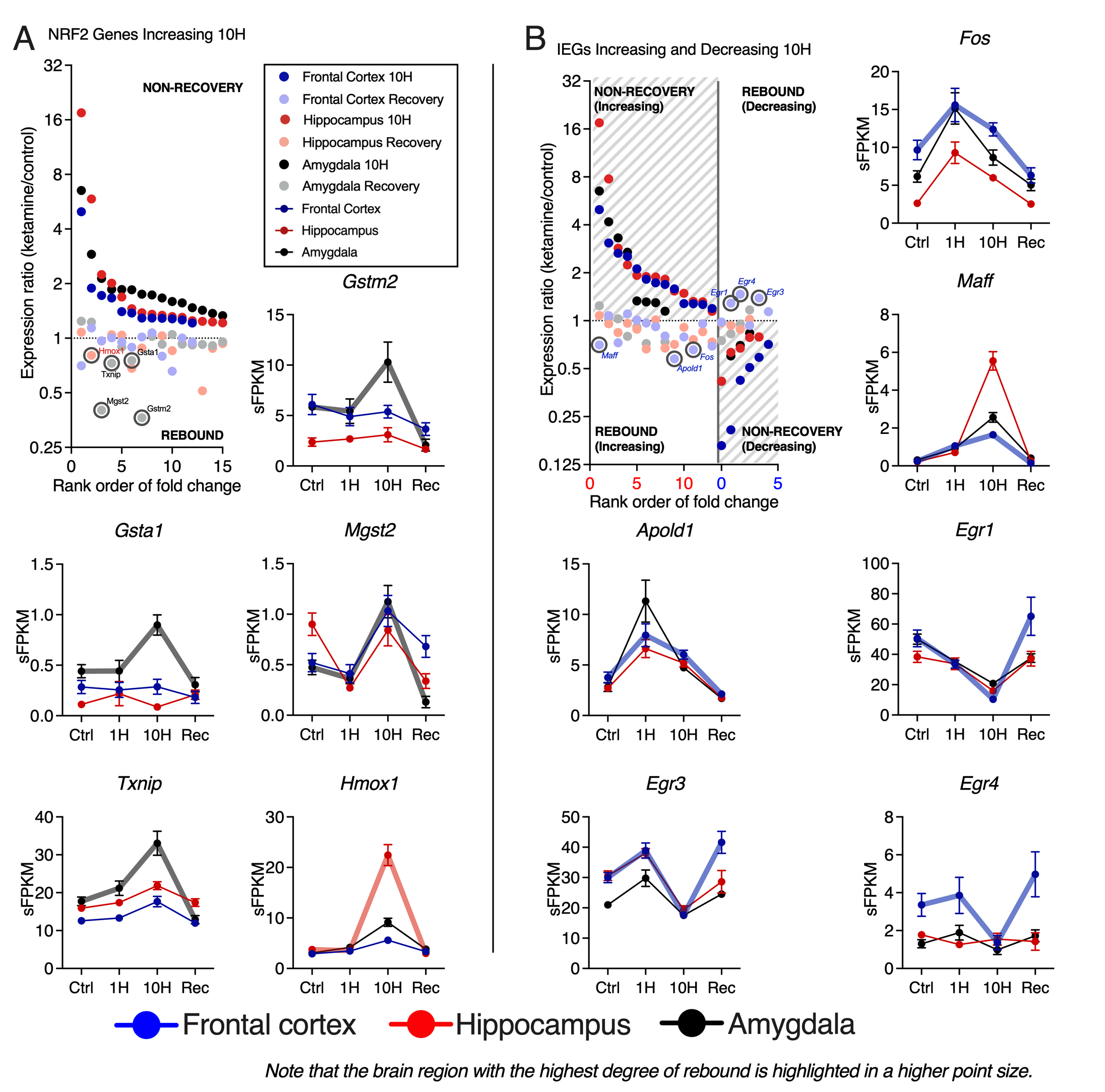


**Supplementary Figure 7. Quantification of rebound effects in the recovery relative to the 10hr gene changes for genes in the Nrf2 pathways and IEG pathways** Frontal cortex is plotted in blue; Hippocampus in red; Amygdala in black. **A.** Genes involved in the Nrf2 oxidative stress response pathway and increasing at 10hr were ranked in order of their 10hr expression ratio and plotted alongside the gene expression ratios at recovery. Genes below the dotted line at 1 (no change) are rebounding, whereas those above it are trending towards non-recovery. Specific genes with high degrees of rebound in a specific brain region were separated out and graphed for their expression across the time course in all three regions to illustrate this effect at the individual gene level. Of such rebounding genes, four of the five selected show a trend towards rebound in the amygdala. **B.** A similar graph was produced for immediate early genes (IEGs) and related genes. In this plot, both increasing and decreasing genes are shown, with increasing genes plotted on the x-axis first (in red ticks) and decreasing genes plotted on an adjoining x-axis (blue ticks, separated by vertical line). On the left are the genes increasing at 10hr, and on the right are those decreasing at 10hr. For the increasing genes, rank order was determined in terms of descending expression ratio value. Rank order was reset for the decreasing genes in terms of ascending expression ratio value, as can be observed in the jump from 1 to 0.125 at the boundary between increasing and decreasing IEGs. Of the rebounding IEGs separated out, all six show a trend towards rebound in the frontal cortex. Notably, some of the same genes appear in Supplementary Figure 5, because some of these genes are among those that demonstrate the highest levels of rebound in the dataset. Note that in panel graphs the brain region showing the greatest degree of rebound is shown with a higher point size connecting line for emphasis.

**
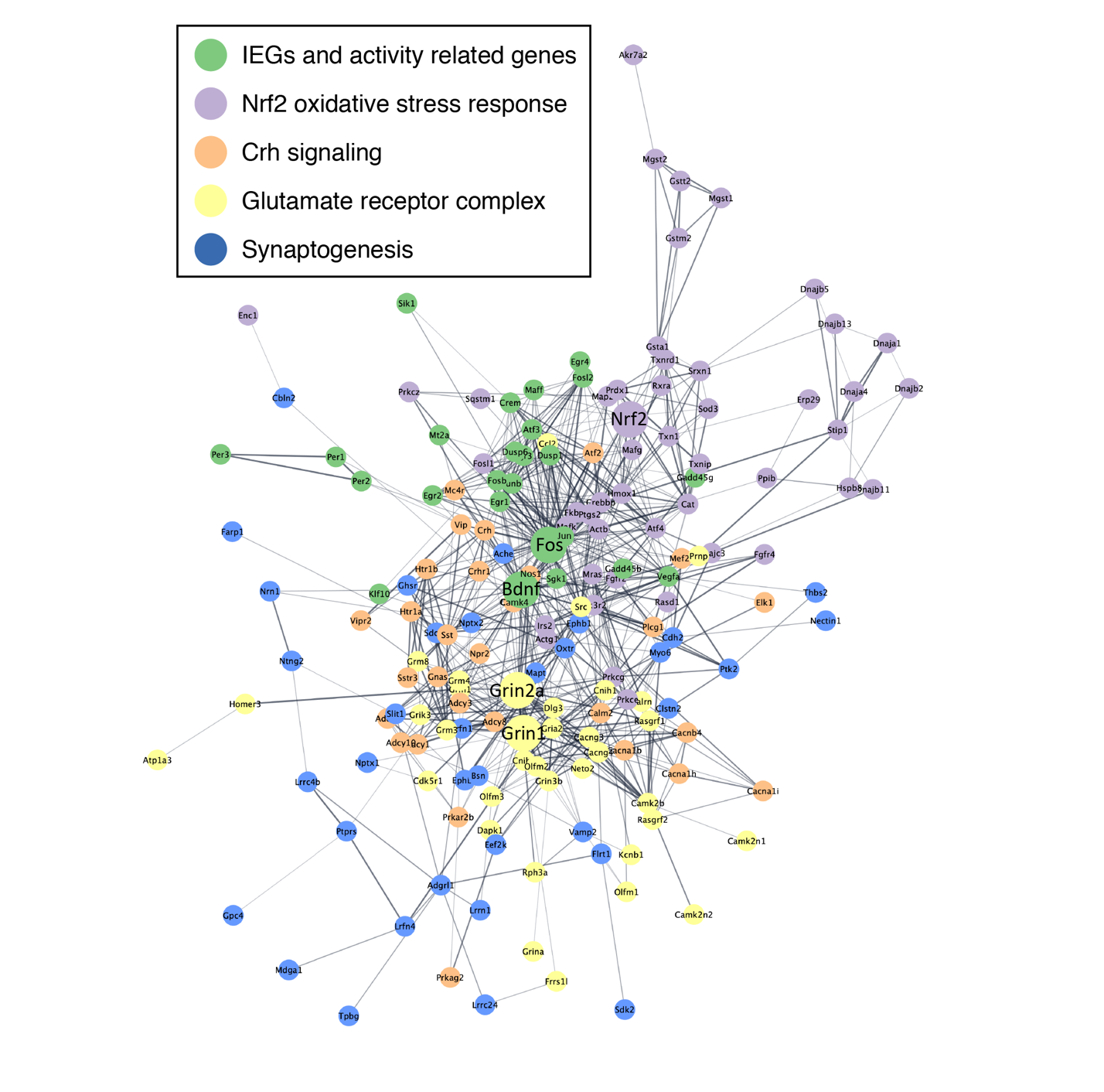
**

**Supplementary Figure 8. Protein-protein interaction network for genes in the canonical pathway analysis.** The list of genes identified in any of the five core pathways discussed in the paper (Figure 3) were analyzed for protein-protein interactions. Analysis was performed using the STRING database (string-db.org) without the addition of interactors from outside the present dataset, and following the standard parameters (Szklarczyk et al., 2019). The resultant interaction network was colored according to pathway in Cytoscape and Adobe Illustrator with the central nodes visualized using a larger circle size for emphasis. This method was also used in Figure 4D following the same parameters. The pathways are generally interconnected, as indicated by the presence of one central mass of interconnected proteins. However, several genes within some pathways, such as the Nrf2 pathway (purple) appear to have more connections to each other than to genes in other pathways. Within this interaction network, several of the central nodes included Nrf2, Fos, Bdnf, and the NMDA receptor subunits Grin1 and Grin2a, which are generally either broad regulators of several of these pathways, or indeed the main pharmacological target of ketamine (the NMDA receptor). Multiple proteins for biogenic amine and neuropeptide receptors or neuropeptides themselves are connected with proteins in the Crh pathway. Multiple proteins in the Fos, Jun and activating transcription factor leucine zipper families are connected to genes in the activity-related pathway, as well as the circadian regulated period (Per) transcriptional repressor proteins. These pathways are discussed in the paper as distinct, but are all modulated by ketamine administration, and are apparently connected by interaction across pathways.


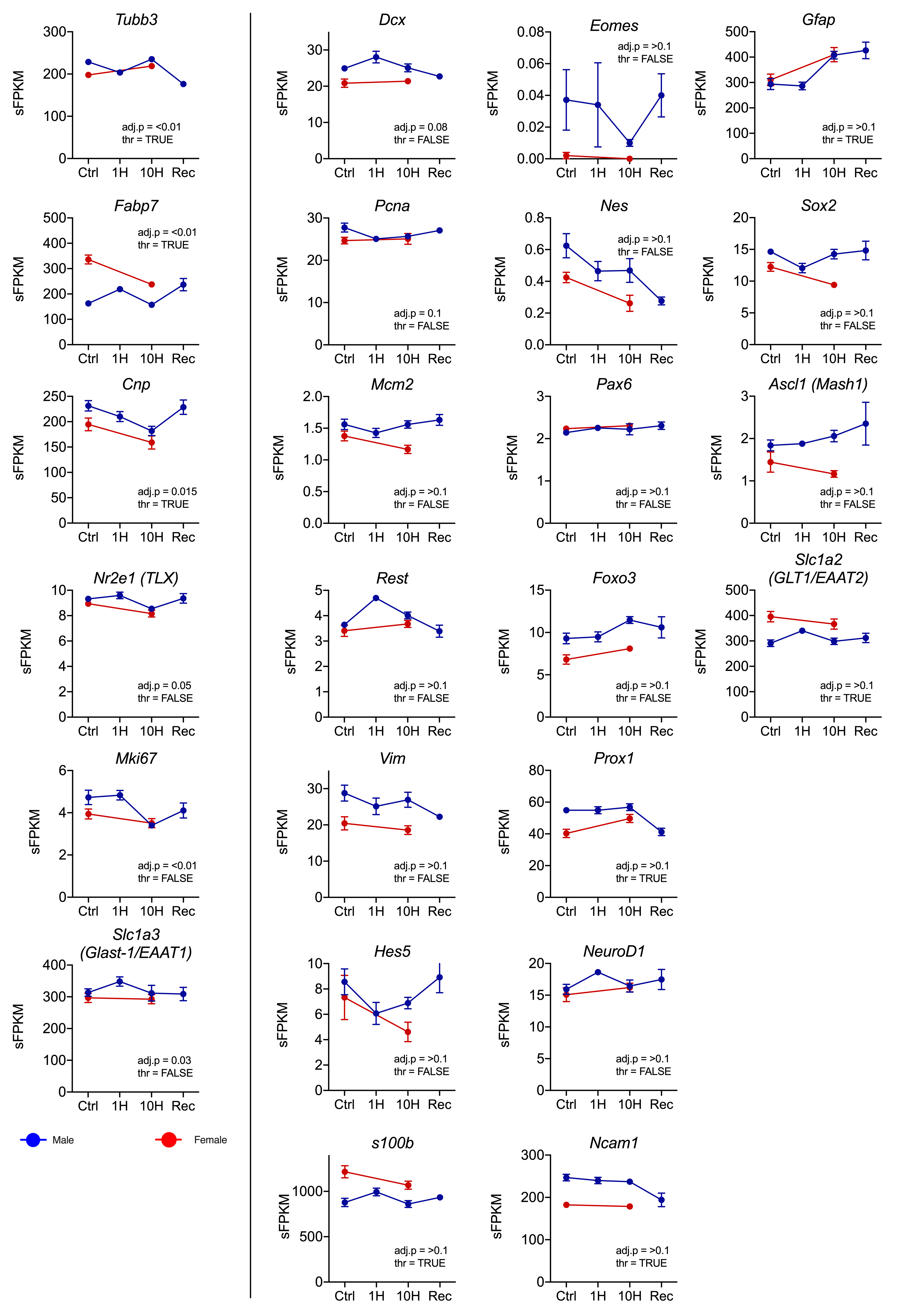


**Supplementary Figure 9. Panel of hippocampal genes related to neurogenesis.** Neurogenesis has been proposed as a mechanism of ketamine action (Clarke et al., 2017). Based on the proposition that ketamine infusion leads to increased hippocampal neurogenesis underlying the sustained antidepressant effects of this intervention, we looked for transcriptomic signatures of neurogenesis induction in the hippocampus in males and females. Males are shown in blue; females in red. These markers were selected from literature reviews and include some genes that are non-specific markers for the phenomenon (Zywitza et al., 2018, Zhang and Jiao, 2015). These genes and their full gene names are summarized in Supplementary Table 7. The majority of these genes were either not changing or significantly reduced. Notably, *Gfap* was significant and shows sustained elevation, and could be a marker of an early phase of neurogenesis (Garcia et al., 2004). However, this marker is not specific for neurogenesis, and is also a widely-used marker of mature astrocytes, which contribute much more to the overall *Gfap* signal detected with RNA-Seq of tissue homogenates. The lack of effect on transcription of these markers is also consistent with previous findings showing that ketamine could restore atrophied spines that might otherwise be degraded rather than stimulate generation of new outgrowths (Moda-Sava et al., 2019).

**
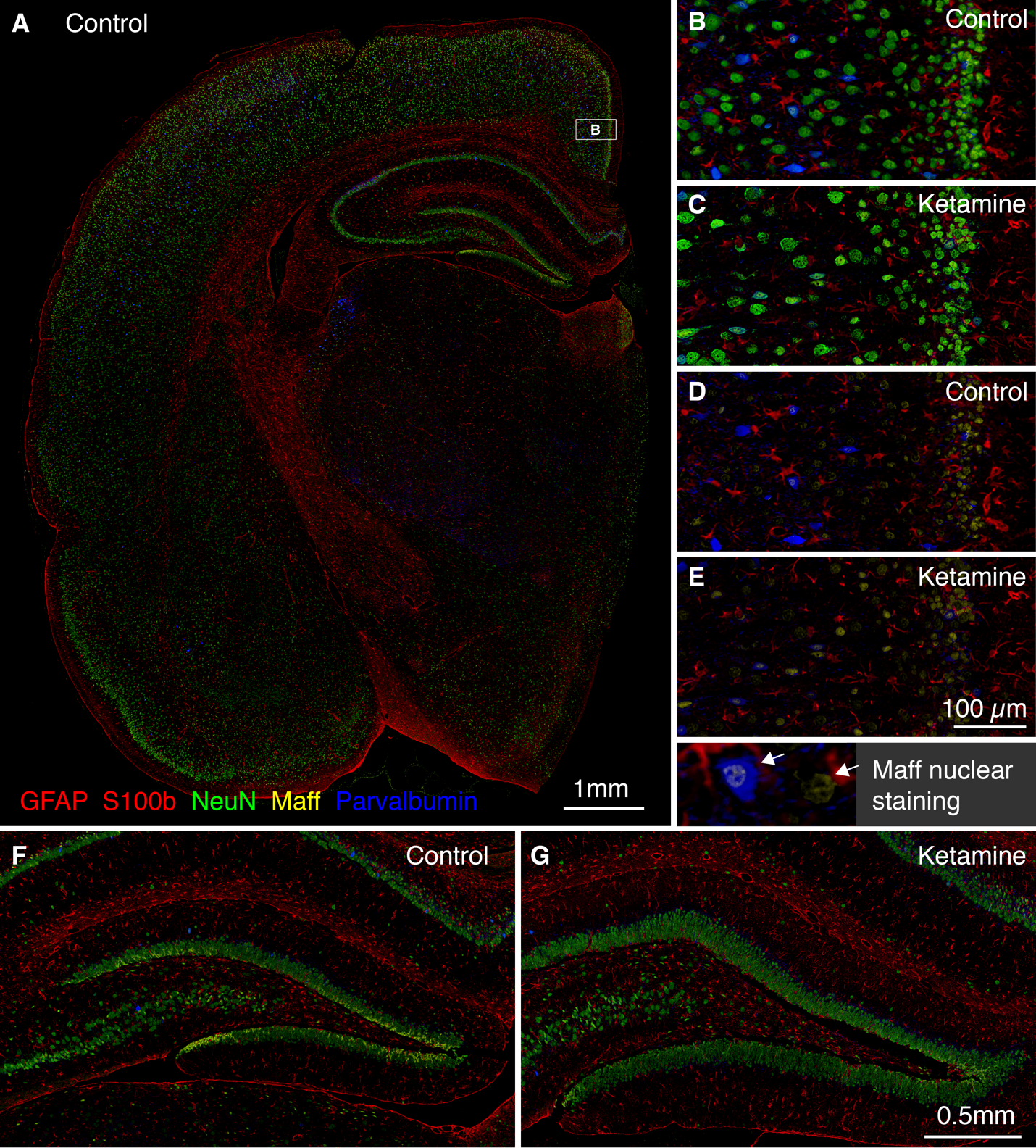
**

**Supplementary Figure 10. Immmunohistochemical staining for Gfap, S100-beta, NeuN, parvalbumin, and Maff proteins.** Antibodies to each of these proteins were used with fluorescent secondary detection and scanned as described in the section on multiplex fluorescent in situ hybridization. Microscopy methods and sections are identical to those described elsewhere in the manuscript. Control and 10hr ketamine animals were compared using this method to examine changes in protein distribution and intensity of immunoreactivity. Based on the finding that *Gfap* mRNA was induced by ketamine, we investigated GFAP protein. The Maff protein was included as well because it shows a strong induction transcriptionally, and it is unclear what cell type produces the transcript. **A.** Whole hemibrain sections (N=3) were stained for this multiplex combination of antibodies. GFAP and s100b are shown in the same channel for visualization, as they detect different structural proteins within astrocytes. **B, C.** An enlargement is shown within medial cortex, comparable to where *Bdnf* and *Fos* mRNA was induced. Despite the strong induction of Maff message, there was no consistent and apparent induction of Maff protein, nor differences in GFAP immunostaining. Maff staining was localized within the nucleus and cytoplasm, with some cells showing relatively specific nuclear staining, and others broadly immunoreactive for Maff antibody. **D, E.** The same region is shown without the green channel (NeuN) to better visualize Maff and GFAP staining. **F, G.** A high-resolution image of the hippocampus is included, but showed no apparent differences between control and ketamine exposed animals similar to other brain regions. Potentially, the large amount of GFAP present at the protein level makes the changes at the mRNA level difficult to see at the whole protein level. This protein turns over slowly, being a major structural protein in astrocytes.

The following antibodies were used in this experiment:

Rabbit IgG anti-MAFF (ProteinTech, cat# 12771-1-AP)

Chicken IgY anti-NeuN (Millipore Sigma, cat# ABN91)

Mouse IgG1 anti-Parvalbumin (Millipore Sigma, cat# MAB1572)

Mouse IgG2a anti-S100b (Millipore Sigma, cat# MAB079-1)

Mouse IgG2b anti-GFAP (BD Biosciences, cat# 556330)


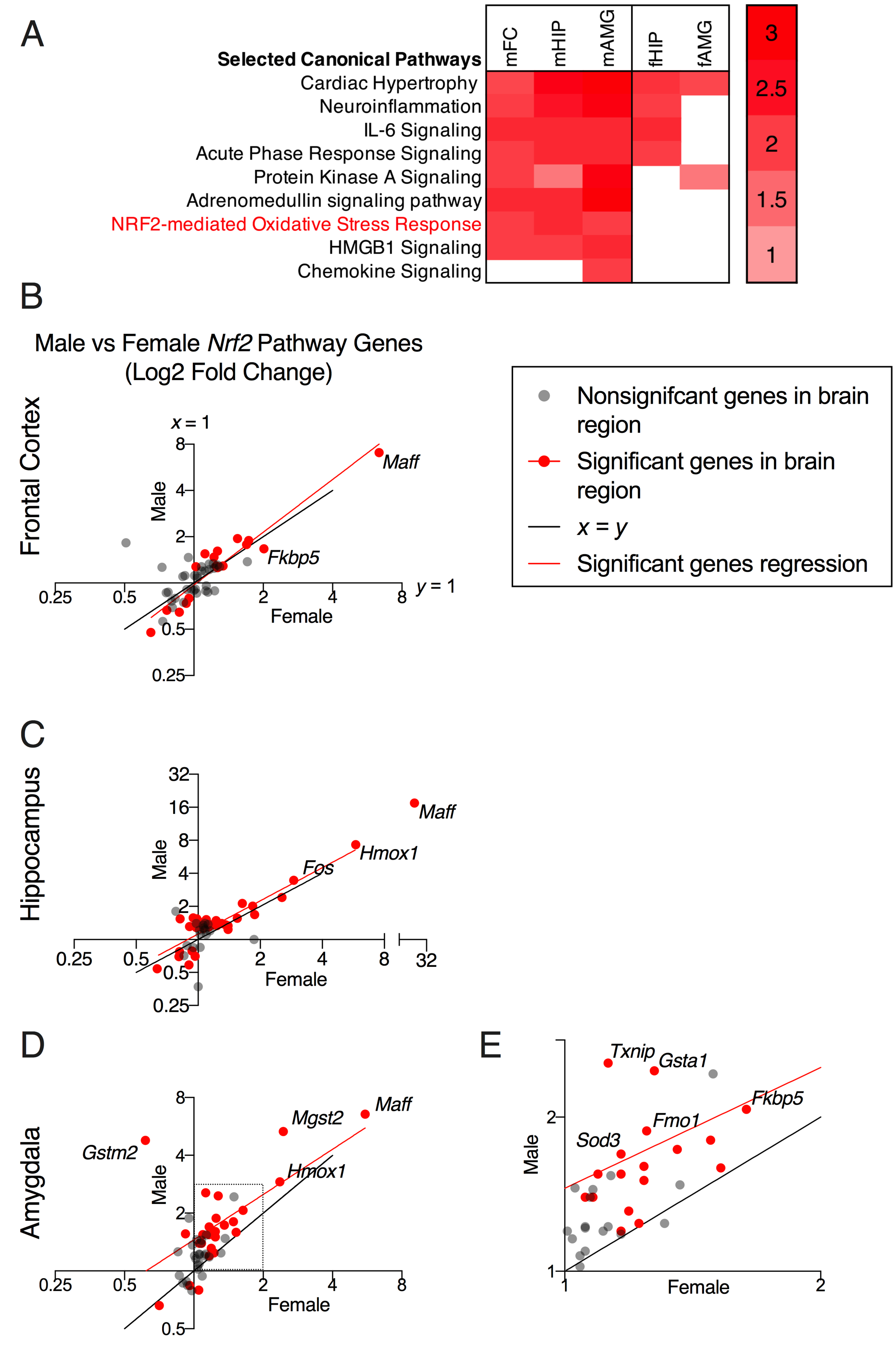


**Supplementary Figure 11. Canonical pathway enrichment analysis for sexually dimorphic gene clusters in amygdala dataset and examination of correlation between male and female transcriptional datasets for significant Nrf2 pathway genes in each brain region.** In Figure 2, two clusters (gold and blue) show a tendency towards sexual dimorphism specifically in the amygdala. In addition to examining the cellular enrichment of those genes, we performed pathway enrichment analysis using Ingenuity Pathway Analysis software (Qiagen). **A.** An overall trend towards greater induction of most pathways was observed in male animals. Of note, while the male-specific enrichment was based on generally low numbers of genes, the algorithm also reported greater predicted activation of the Nrf2 pathway in male animals relative to females (highlighted in red). The heatmap shows the activation Z-scores reported by IPA for the pathways listed which are used for predicting whether a pathway or function is activated or inhibited. Note that all Z-scores are positive for pathway activation, which is also reflected in the absence of negative values on the flame scale. **B**,**C**,**D,E**. Based on the prediction from IPA software and the general trend towards greater induction in some datasets in the male animals relative to females (e.g., Figure 3), we specifically examined the Nrf2 pathway with respect to sex differences in correlation plots for each brain region. Log2 expression ratios (ER) for male (y-axis) and female (x-axis) rats are plotted against each other. Sex concordance is denoted by the gray line (male log2ER = female log2ER) denoting equal change in expression in both sexes. All genes in the Nrf2 pathway that are significant are plotted for each brain region (gray dots) with the genes significant in at least one sex in the brain region for which data are plotted shown in red. **B.** Within the frontal cortex, activated genes in the Nrf2 pathway were highly correlated showing similar degrees of induction in both sexes. For female cortex, data were obtained from 2 rats. **C.** In the hippocampus no indication of between sex differences in the Nrf2 pathway was observed and, like frontal cortex, genes from both sexes showed comparable induction. **D.** Among significant Nrf2 pathway genes in the amygdala, there was a trend towards a greater fold change in the male animals relative to females denoted by the upwards shift in the red line. Selected genes showing the greatest degree of sexual dimorphism are labeled, including the highly significant gene *Maff* which codes for a basic leucine zipper transcription factor that lacks a transactivation domain, and Mgst2, involved in leukotriene C4 biosynthesis. **E.** An enlarged view of the points in the center of D are shown, which show the same overall pattern and allowed for the labeling of additional genes such as *Fkbp5* which is implicated in several pathways in the current analysis.

**
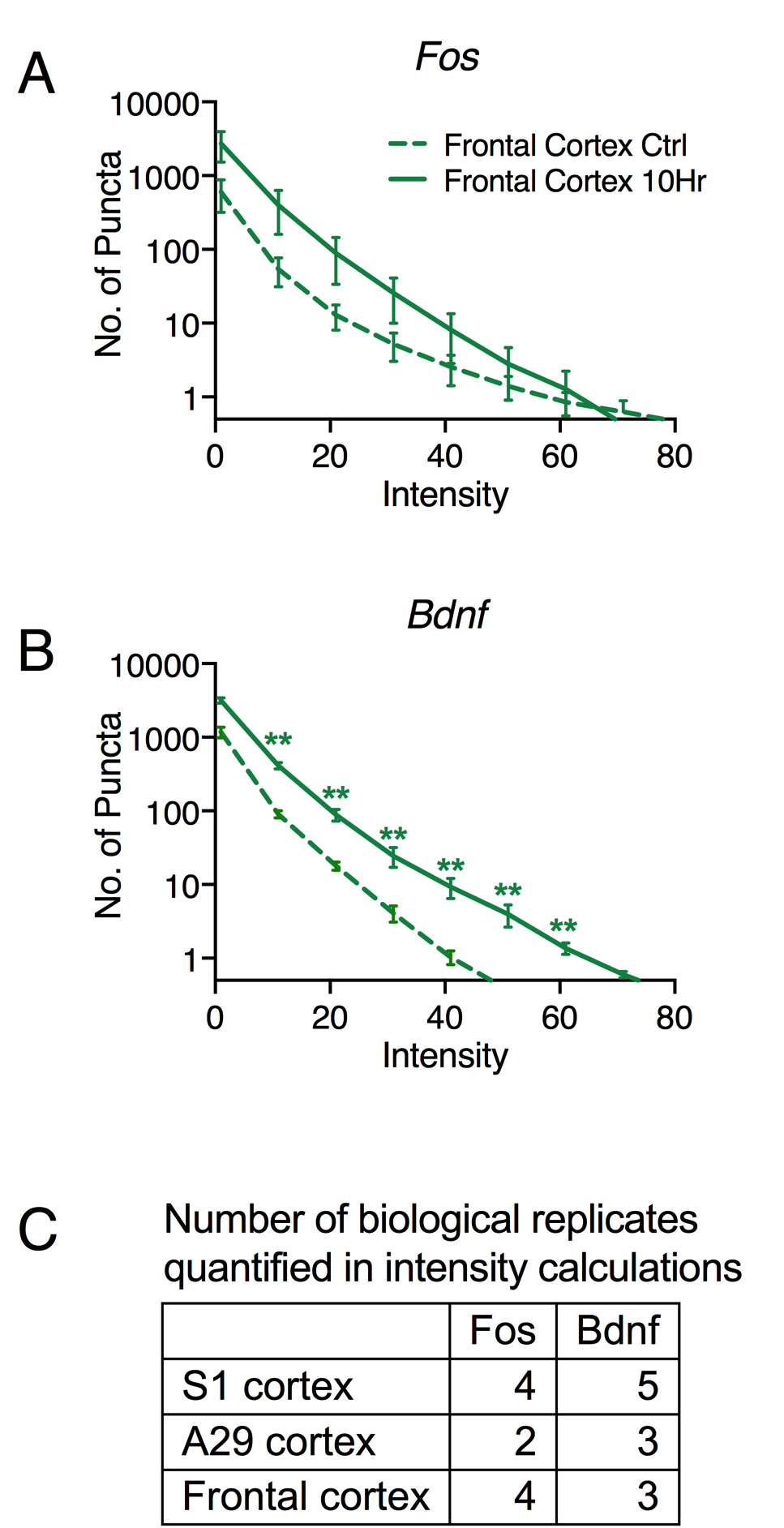
**

**Supplementary Figure 12. *In situ* hybridization of *Fos* and *Bdnf* in frontal cortex.** The evaluations shown in Figure 5 were expanded to include a region of frontal cortex similar to that which was analyzed using RNA-Seq. **A, B.** The changes in this region were less pronounced in general, with *Bdnf*, but not *Fos* showing a significant increase in the intensity of the measurement. **C.** A table of the number of biological replicates in each staining experiment is shown. The control and 10hr ketamine numbers are generally the same because a control and ketamine sample was stained and scanned on the same slide for all evaluations.

**
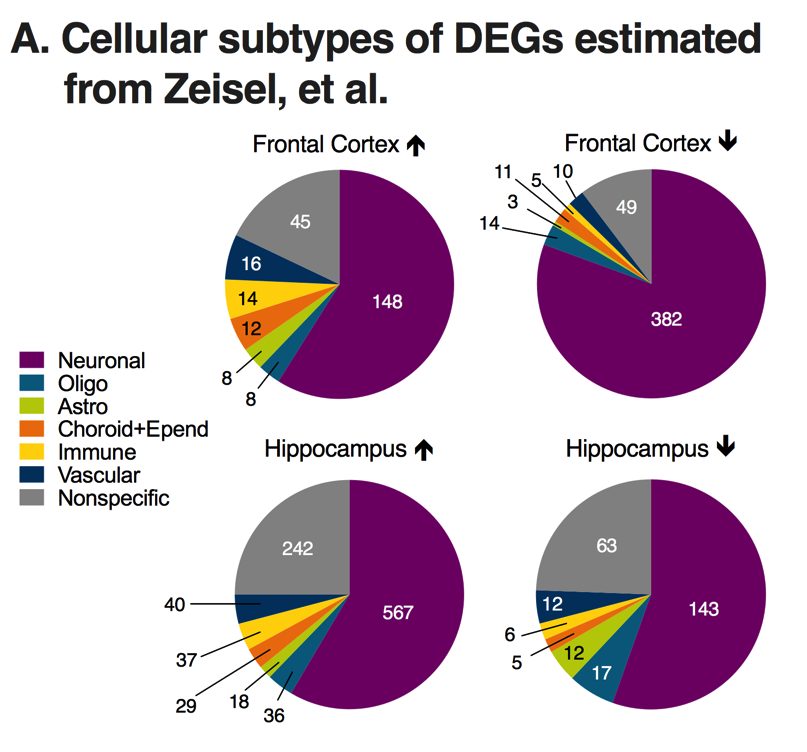
**

**Supplementary Figure 13. Cellular sybtypes of differentially expressed genes estimated using the dataset from Zeisel, et al. 2014.** Similar to the analysis in Figure 4, a second dataset was queried to estimate cellular origin of differentially expressed genes. This second dataset consisted of a single-cell sequencing experiment from mouse cortex and hippocampus.(Zeisel et al., 2015). Specifically, the differentially expressed genes from frontal cortex and hippocampus were mapped to the data in Zeisel et al., and the molecules/cell expression data from Zeisel et al. was used to create the above pie chart. Note that in some cases subcellular populations were pooled to examine broad categories such as “neuronal” or “microglia” despite the original dataset having finer gradations. Differentially expressed genes from the amygdala were not examined in Zeisel because their single cell experiment as focused on cortex and hippocampus.
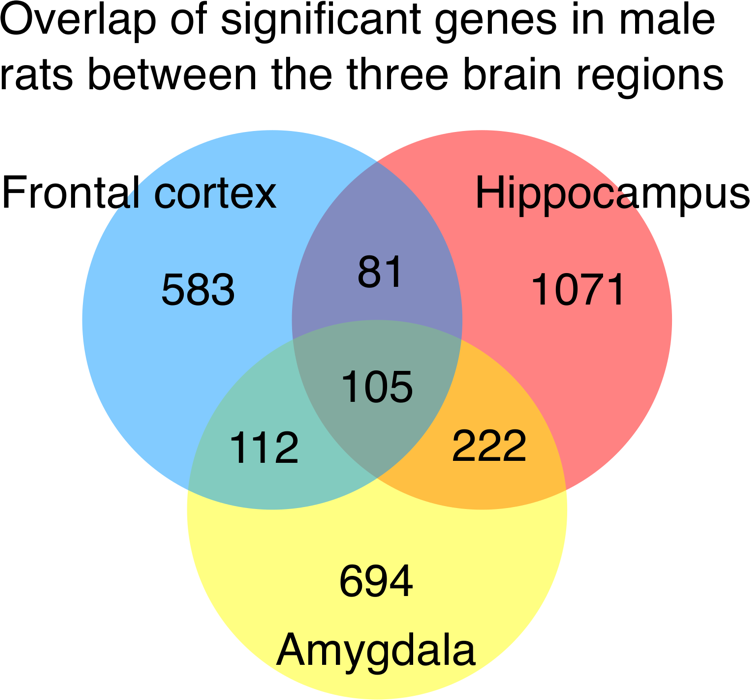


**Supplementary Figure 14. Venn diagram of overlapping significant gene lists between the three male datasets.** Genes that met statistical threshold in the study were compared for the three male datasets. The lists of genes in each category are shown in Supplementary Table 8.

**REFERENCES**

CLARKE, M., RAZMJOU, S., PROWSE, N., DWYER, Z., LITTELJOHN, D., PENTZ, R., ANISMAN, H. & HAYLEY, S. 2017. Ketamine modulates hippocampal neurogenesis and pro-inflammatory cytokines but not stressor induced neurochemical changes. *Neuropharmacology,* 112**,** 210-220.

GARCIA, A. D., DOAN, N. B., IMURA, T., BUSH, T. G. & SOFRONIEW, M. V. 2004. GFAP-expressing progenitors are the principal source of constitutive neurogenesis in adult mouse forebrain. *Nat Neurosci,* 7**,** 1233-41.

MODA-SAVA, R. N., MURDOCK, M. H., PAREKH, P. K., FETCHO, R. N., HUANG, B. S., HUYNH, T. N., WITZTUM, J., SHAVER, D. C., ROSENTHAL, D. L., ALWAY, E. J., LOPEZ, K., MENG, Y., NELLISSEN, L., GROSENICK, L., MILNER, T. A., DEISSEROTH, K., BITO, H., KASAI, H. & LISTON, C. 2019. Sustained rescue of prefrontal circuit dysfunction by antidepressant-induced spine formation. *Science,* 364.

SINGER, R. H. & PENMAN, S. 1972. Stability of HeLa cell mRNA in actinomycin. *Nature,* 240**,** 100-2.

SINGER, R. H. & PENMAN, S. 1973. Messenger RNA in HeLa cells: kinetics of formation and decay. *J Mol Biol,* 78**,** 321-34.

SZKLARCZYK, D., GABLE, A. L., LYON, D., JUNGE, A., WYDER, S., HUERTA-CEPAS, J., SIMONOVIC, M., DONCHEVA, N. T., MORRIS, J. H., BORK, P., JENSEN, L. J. & MERING, C. V. 2019. STRING v11: protein-protein association networks with increased coverage, supporting functional discovery in genome-wide experimental datasets. *Nucleic Acids Res,* 47**,** D607-D613.

ZEISEL, A., MUNOZ-MANCHADO, A. B., CODELUPPI, S., LONNERBERG, P., LA MANNO, G., JUREUS, A., MARQUES, S., MUNGUBA, H., HE, L., BETSHOLTZ, C., ROLNY, C., CASTELO-BRANCO, G., HJERLING-LEFFLER, J. & LINNARSSON, S. 2015. Brain structure. Cell types in the mouse cortex and hippocampus revealed by single-cell RNA-seq. *Science,* 347**,** 1138-42.

ZHANG, J. & JIAO, J. 2015. Molecular Biomarkers for Embryonic and Adult Neural Stem Cell and Neurogenesis. *Biomed Res Int,* 2015**,** 727542.

ZYWITZA, V., MISIOS, A., BUNATYAN, L., WILLNOW, T. E. & RAJEWSKY, N. 2018. Single-Cell Transcriptomics Characterizes Cell Types in the Subventricular Zone and Uncovers Molecular Defects Impairing Adult Neurogenesis. *Cell Rep,* 25**,** 2457-2469 e8.
